# Phylogeny of Hawaiian *Melicope* (Rutaceae): RAD-seq Resolves Species Relationships and Reveals Ancient Introgression

**DOI:** 10.3389/fpls.2019.01074

**Published:** 2019-09-17

**Authors:** Claudia Paetzold, Kenneth R. Wood, Deren A. R. Eaton, Warren L. Wagner, Marc S. Appelhans

**Affiliations:** ^1^Department of Systematics, Biodiversity and Evolution of Plants (with Herbarium), University of Göttingen, Goettingen, Germany; ^2^National Tropical Botanical Garden, Kalaheo, HI, United States; ^3^Department of Ecology, Evolution and Environmental Biology, Columbia University, New York, NY, USA; ^4^Department of Ecology, Evolution, and Environmental Biology, Columbia University, New York, NY, United States; ^5^Department of Botany, Smithsonian Institution, Washington, DC, United States

**Keywords:** adaptive radiation, *D*-statistics, Hawaiian flora, introgression, Marquesas Islands, Quartet Sampling, RAD-seq, Rutaceae

## Abstract

Hawaiian *Melicope* are one of the major adaptive radiations of the Hawaiian Islands comprising 54 endemic species. The lineage is monophyletic with an estimated crown age predating the rise of the current high islands. Phylogenetic inference based on Sanger sequencing has not been sufficient to resolve species or deeper level relationships. Here, we apply restriction site-associated DNA sequencing (RAD-seq) to the lineage to infer phylogenetic relationships. We employ Quartet Sampling to assess information content and statistical support, and to quantify discordance as well as partitioned ABBA-BABA tests to uncover evidence of introgression. Our new results drastically improved resolution of relationships within Hawaiian *Melicope*. The lineage is divided into five fully supported main clades, two of which correspond to morphologically circumscribed infrageneric groups. We provide evidence for both ancestral and current hybridization events. We confirm the necessity for a taxonomic revision of the *Melicope* section *Pelea*, as well as a re-evaluation of several species complexes by combining genomic and morphological data.

## Introduction

Oceanic islands have long been a focal point of evolutionary studies, as they represent a microcosm for examining the process of speciation. This microcosm is shaped by a combination of factors: (1) islands are geographically small and discrete units, sometimes far removed from continental landmasses; (2) colonizations or secondary arrivals are relatively infrequent, and thus, gene flow between the source areas and island systems is restricted; and (3) islands often have dynamic geological histories that give rise to extensively varying landscapes with numerous ecological niches (Emerson, 2002; Price and Wagner, 2018). These factors can often lead to high levels of endemism, which is often the result of adaptive radiation of a limited number of colonizers (Price and Wagner, 2004; Losos and Ricklefs, 2009; Keeley and Funk, 2011). Synthesizing the unique aspects of island evolution and extrapolating results to larger scales may allow us to better uncover general patterns and processes in evolution. Such phenomena include identifying factors affecting successful colonization and adaptive radiation (Carlquist, 1967; Carlquist, 1974; Paetzold et al., 2018), morphological or ecological shifts (e.g., “insular woodiness”; Carlquist, 1974; Lens et al., 2013), the spatiotemporal origins of lineages (Appelhans et al., 2018a), reconstructing colonization events (Harbaugh et al., 2009), and studying co-evolution (Roderick, 1997). These insights may result in further questions regarding taxonomy, species richness, medicinal or technical applications, and conservation (e.g., Francisco-Ortega et al., 2000).

Adaptive radiations on islands are of special interest for connecting changes in morphology and ecology through time (Givnish, 1998) but require well-resolved phylogenies to do so. In the Hawaiian Islands, phylogenetic studies based on morphology and taxonomy have sometimes overestimated the number of colonization events, because high levels of morphological diversity led researchers to overestimate lineage diversity and the number of colonization events (Price and Wagner, 2018). In contrast, molecular phylogenetic studies have revealed that many enigmatic Hawaiian plant radiations colonized the islands only once followed by adaptive radiation: the Hawaiian lobeliads (Campanulaceae; Givnish et al., 2009), *Psychotria* (Rubiaceae; Nepokroeff et al., 2003), *Silene* (Caryophyllaceae; Eggens et al., 2007), *Touchardia/Urera* (Urticaceae; Wu et al., 2013), and *Melicope* (Harbaugh et al., 2009; Appelhans et al., 2014a). Polyploidization and hybridization events were also discovered to predate colonization and radiation in several island lineages, including the Hawaiian silverswords (Asteraceae; Baldwin and Sanderson, 1998; Barrier et al., 1999) and mints (Lamiaceae; Roy et al., 2015) along with the Pan- Pacific sandalwoods (Santalaceae; Harbaugh, 2008), suggesting evolutionary success in young hybrid or polyploid colonists (Carr, 1998; Paetzold et al., 2018).

Time-scaled phylogenies have revealed that most Hawaiian radiations are ≤5 Myr old, which corresponds to the age of the oldest current main islands, Kauaʻi and Niʻihau. This suggests a bottleneck for dispersal from older (and now largely submerged) leeward islands to the current main islands. However, there are several known exceptions of lineages older than 5 Myr, including *Drosophila*, damselflies, lobeliads, *Zanthoxylum* (Rutaceae), as well as *Melicope* (Price and Clague, 2002; Keeley and Funk, 2011; Appelhans et al., 2018a; Appelhans et al., 2018b). Most phylogenetic studies of Hawaiian flora, however, have relied on few sequenced loci and have thus lacked sufficient power to resolve recent rapid radiations where hybridization, incomplete lineage sorting (ILS), and polyploidy may be common. Newer genomic tools are likely to provide more accurate estimates that may transform our understanding of island radiations.

The genus *Melicope* comprises about 235 species of shrubs and trees distributed throughout SE Asia and Australasia, extending to the Mascarene Islands and Madagascar in the West and most of the Pacific Archipelagos in the East (Hartley, 2001). There are 54 species of *Melicope* endemic to the Hawaiian Islands (Appelhans et al., 2017; Wood et al., 2017), 41 of which are single island endemics (Stone et al., 1999). Hawaiian *Melicope* were initially placed in the genus *Pelea* together with species from the Marquesas Islands (Stone, 1969; Stone et al., 1999) but later incorporated into *Melicope*, forming the majority of the section *Pelea* (Hartley, 2001). Hawaiian *Pelea* was divided into four sections based mainly on the grade of carpel connation: *Apocarpa*, *Cubicarpa*, *Megacarpa*, and *Pelea*. Since the incorporation of the genus *Pelea* into *Melicope*, these sections have not been formally recognized within the larger infrageneric taxonomy for *Melicope* as recognized by Hartley (2001) but are still being used informally as species groups (Appelhans et al., 2014a), and we refer to them as Stone’s sectional species groups (Stone’s sections) from here on. The most current and comprehensive taxonomic treatment of Hawaiian *Melicope* was considered “provisional” by the authors (Stone et al., 1999), as species boundaries are difficult to define in some cases. Examples include three described species complexes, where the incorporated species vary from each other primarily in the degree of fruit pubescence; the *Melicopeelliptica* complex based mainly in Oʻahu (six species), the Hawaiian-based *Melicopevolcanica* complex (four species), and the Kauaʻi-based *Melicopekavaiensis* complex (five species) (Stone et al., 1999).

In contrast to other successful island radiations, the colonization of the Hawaiian Archipelago in *Melicope* was not preceded by a recent polyploidization event. In general, the genus *Melicope* shows a uniform chromosome number (Paetzold et al., 2018). To date, phylogenetic relationships in Hawaiian *Melicope* have been investigated in four molecular studies (Harbaugh et al., 2009; Appelhans et al., 2014a; Appelhans et al., 2014b; Appelhans et al., 2018a), with a combination of up to six nuclear and plastid genomic regions amplified using polymerase chain reaction. Hawaiian *Melicope* was shown to be derived from a single colonization event (Harbaugh et al., 2009). The origin of the lineage was dated to the Mid or Late Miocene (Appelhans et al., 2018a), predating the age of Kauaʻi and Niʻihau (Price and Clague, 2002). In addition, the Hawaiian endemic genus *Platydesma* is nested within *Melicope* as a monophyletic sister group to the Hawaiian species and has since been reduced (Appelhans et al., 2017). Statistically supported incongruences between individual genomic regions were not observed, yet the resolution of relationships within and among the clades was in general medium to poor (Harbaugh et al., 2009; Appelhans et al., 2014a; Appelhans et al., 2014b; Appelhans et al., 2018a). However, two independent Hawaiian origins of the Marquesan *Melicope* radiation, which encompasses seven species, were inferred (Appelhans et al., 2014a; Appelhans et al., 2014b; Appelhans et al., 2017).

Restriction site-associated DNA sequencing (RAD-seq; Miller et al., 2007; Baird et al., 2008) is among the most frequently used reduced representation methods employed in plant systematics. To date, most phylogenetic RAD-seq studies have focused mostly on populations or closely related species (Ree and Hipp, 2015; Díaz-Arce et al., 2016; Hodel et al., 2017). However, a simulated RAD investigation in *Drosophila* revealed the method to be potentially applicable in groups aged up to 60 Myr (Rubin et al., 2012). Since then, application to deeper species-level relationships has increased (e.g., Eaton and Ree, 2013; Hipp et al., 2014; Eaton et al., 2017), facilitated by the development of RAD-seq assembly pipelines targeted at phylogenetic research (Eaton, 2014).

Incongruence between datasets has been a long-standing occurrence in molecular phylogenetic inference, traditionally manifesting as incongruences between different gene trees. The advance of next-generation sequencing (NGS) technology has shown that the issue is not solved by merely incorporating more data (Jeffroy et al., 2006). There are three possible categories of confounding information in a phylogenetic study: noise, systematic error, and an underlying biological signal. Noise is an effect of the inherently stochastic nature of sequence evolution and leads to a deterioration of phylogenetic signal over time. As such, noise most heavily impacts very small datasets and deep nodes (Misof et al., 2014). Incongruence may also reflect a true biological signal, for example, the presence of ILS or non-tree-like evolution, i.e. introgression, hybridization, or recombination (Misof et al., 2014; Salichos et al., 2014). Effects of hybridization range from introgression of individual alleles, to organelle capture, to hybrid speciation (Currat et al., 2008; Stegemann et al., 2012; Twyford and Ennos, 2012). Either of these processes will result in discordant gene trees, and several approaches have been proposed to unravel them. Based on the distributions of conflicting phylogenetic patterns in the genome, it is possible to distinguish the more stochastic signal of ILS from the directional and asymmetric signal of hybridization (Durand et al., 2011).

Here, we apply RAD-seq to Hawaiian *Melicope*, a lineage with a crown age of ca. 10 Myr (Appelhans et al., 2018a). We use RAD-seq to infer species-level relationships in the lineage, in a phylogenetic context of several colonization events of individual islands, multiple possible bottlenecks, and adaptive radiations within a lineage. The taxonomic implications of our phylogenetic results are discussed within the framework of evidence for both ancient and current introgression.

## Materials and Methods

### Taxon Sampling

**Table 1** details the identity and origin of the 101 samples of this study: 6 outgroup and 95 ingroup specimens representing 41 Hawaiian species (81% of the lineage). Two samples represent the two independent colonization events to the Marquesas Islands (28% of Marquesan species). Taxonomic treatment follows species recognized in Wood et al. (2016) plus a recently described species (Wood et al., 2017) and including *Platydesma* (Appelhans et al., 2017). Additionally, morphologically divergent specimens of *Melicope barbigera* (KW16722 and KW16718) and *Melicope ovata* (KW16762, KW17082, and MA663) were included (**Table 1**, asterisk) to elucidate whether these might represent separate taxa. We also included two specimens, KW17111 and KW15733, which correspond closely, though not entirely, to the description of *Melicope wawraeana* as delimited by Stone et al. (1999). Even the Oʻahu populations that were considered the core of *M*.*wawraeana* are variable, suggesting that it is a potentially artificial taxon (Stone et al., 1999). Since the morphology of the two specimens did not correspond entirely to the Oʻahu populations considered to be *M*.*wawraeana*, we included them here as *Melicope* sp. (**Table 1**).

**Table 1:** Samples within this study including origin, voucher placement, and assignment to Stone’s sections.

Species	Stone’s section	Collection number, Herbarium voucher	Origin
***Melicope adscendens*** **(H. St. John & E. P. Hume) T. G. Hartley & B. C. Stone**	***Apocarpa***	**Appelhans MA628 (silica sample only, ORPF)**	**Maui**
*Melicope anisata* (H. Mann) T. G. Hartley & B. C. Stone	*Cubicarpa*	Appelhans MA665 (GOET, PTBG)	Kauaʻi
*M. anisata* (H. Mann) T. G. Hartley & B. C. Stone	*Cubicarpa*	Appelhans MA668 (GOET, PTBG, USA)	Kauaʻi
*Melicope balloui* (Rock) T. G. Hartley & B. C. Stone	*Megacarpa*	Wood KW7685 (PTBG)	Maui
***Melicope barbigera*** **A. Gray**	***Apocarpa***	**Appelhans MA666 (BISH, GOET, PTBG, USA)**	**Kauaʻi**
*M. barbigera* A. Gray	*Apocarpa*	Wood KW15333 (PTBG)	Kauaʻi
*M. barbigera* A. Gray	*Apocarpa*	Wood KW15449 (PTBG)	Kauaʻi
*M. barbigera* A. Gray	*Apocarpa*	Wood KW15961 (PTBG)	Kauaʻi
M. barbigera* A. Gray	*Apocarpa*	Wood KW16722 (PTBG)	Kauaʻi
M. barbigera* A. Gray	*Apocarpa*	Wood KW16718 (PTBG)	Kauaʻi
*Melicope christophersenii* (H. St. John) T. G. Hartley & B. C. Stone	*Megacarpa*	Appelhans MA618 (BISH, GOET, PTBG, USA)	Oʻahu
*Melicope christophersenii* (H. St. John) T. G. Hartley & B. C. Stone	*Megacarpa*	Appelhans MA621 (silica sample only, cultivated at Puʻu Kaʻala)	Oʻahu
*Melicope clusiifolia* (A. Gray) T. G. Hartley & B. C. Stone	*Pelea*	Appelhans MA615 (GOET, PTBG)	Oʻahu
*M. clusiifolia* (A. Gray) T. G. Hartley & B. C. Stone	*Pelea*	Appelhans MA617	Oʻahu
*M. clusiifolia* (A. Gray) T. G. Hartley & B. C. Stone	*Pelea*	Appelhans MA634 (PTBG)	Maui
*M. clusiifolia* (A. Gray) T. G. Hartley & B. C. Stone	*Pelea*	Appelhans MA650 (GOET, PTBG, USA)	Maui
*M. clusiifolia* (A. Gray) T. G. Hartley & B. C. Stone	*Pelea*	Appelhans MA651 (BISH, GOET, PTBG, USA)	Maui
*M. clusiifolia* (A. Gray) T. G. Hartley & B. C. Stone	*Pelea*	Appelhans MA655 (silica sample only)	Maui
*M. clusiifolia* (A. Gray) T. G. Hartley & B. C. Stone	*Pelea*	Appelhans MA657 (GOET, PTBG, USA)	Maui
*M. clusiifolia* (A. Gray) T. G. Hartley & B. C. Stone	*Pelea*	Appelhans MA670	Kauaʻi
*M. clusiifolia* (A. Gray) T. G. Hartley & B. C. Stone	*Pelea*	Appelhans MA672	Kauaʻi
*M. clusiifolia* (A. Gray) T. G. Hartley & B. C. Stone	*Pelea*	Appelhans MA693	Hawaiʻi
*M. clusiifolia* (A. Gray) T. G. Hartley & B. C. Stone	*Pelea*	Appelhans MA695	Hawaiʻi
*M. clusiifolia* (A. Gray) T. G. Hartley & B. C. Stone	*Pelea*	Oppenheimer s.n. (silica sample only)	Maui
***M. clusiifolia*** **(A. Gray) T. G. Hartley & B. C. Stone**	***Pelea***	**Oppenheimer H91641 (US)**	**Lānaʻi**
*M. clusiifolia* (A. Gray) T. G. Hartley & B. C. Stone	*Pelea*	Wood KW16146 (PTBG)	Kauaʻi
***M. clusiifolia*** **(Gray) T. G. Hartley & B. C. Stone**	***Pelea***	**Appelhans MA675**	**Kauaʻi**
*Melicope cornuta* (Hillebr.) Appelhans, K. R. Wood & W. L. Wagner	*Platydesma*	Ching s.n. (silica sample only)	Oʻahu
***M. cornuta*** **var**. ***decurrens*** **(B. C. Stone) Appelhans, K. R. Wood & W. L. Wagner**	***Platydesma***	**Takahama s.n. (silica sample only)**	**Oʻahu**
*Melicope cruciata* (A. Heller) T. G. Hartley & B. C. Stone	*Megacarpa*	Wood KW16251 (PTBG)	Kauaʻi
*Melicope degeneri* (B. C. Stone) T. G. Hartley & B. C. Stone	*Cubicarpa*	Wood KW15903 (PTBG)	Kauaʻi
*M. degeneri* (B. C. Stone) T. G. Hartley & B. C. Stone	*Cubicarpa*	Wood KW15984 (PTBG)	Kauaʻi
*Melicope feddei* (H. Lév.) T. G. Hartley & B. C. Stone	*Megacarpa*	Appelhans MA688 (BISH, GOET, PTBG, USA)	Kauaʻi
*M. feddei* (H. Lév.) T. G. Hartley & B. C. Stone	*Megacarpa*	Wood KW15844 (PTBG)	Kauaʻi
***M. haleakalae*** **(B. C. Stone) T. G. Hartley & B. C. Stone**	***Pelea***	**Appelhans MA645 (BISH, GOET, PTBG)**	**Maui**
*M. haleakalae* (B. C. Stone) T. G. Hartley & B. C. Stone	*Pelea*	Appelhans MA646 (BISH, GOET, PTBG, USA)	Maui
***Melicope haupuensis*** **(H. St. John) T. G. Hartley & B. C. Stone**	***Apocarpa***	**Appelhans MA687 (BISH)**	**Kauaʻi**
*M. haupuensis* (H. St. John) T. G. Hartley & B. C. Stone	*Apocarpa*	Wood KW16791 (PTBG)	Kauaʻi
*M. haupuensis* (H. St. John) T. G. Hartley & B. C. Stone	*Apocarpa*	Wood KW16794 (PTBG)	Kauaʻi
*Melicope hawaiensis* (Wawra) T. G. Hartley & B. C. Stone	*Apocarpa*	Appelhans MA633 (BISH, GOET, PTBG, USA)	Maui
*M. hawaiensis* (Wawra) T. G. Hartley & B. C. Stone	*Apocarpa*	Appelhans MA700	Hawaiʻi
*M. hawaiensis* (Wawra) T. G. Hartley & B. C. Stone	*Apocarpa*	Oppenheimer s.n. (silica sample only)	Maui
*Melicope hiiakae* (B. C. Stone) T. G. Hartley & B. C. Stone	*Megacarpa*	Ching s.n. (silica sample only)	Oʻahu
*Melicope hivaoaensis* J. Florence		Meyer 826	Hivaoa, Marquesas Islands
*Melicope inopinata* J. Florence		Meyer 887	Hivaoa, Marquesas Islands
***Melicope kavaiensis*** **(H. Mann) T. G. Hartley & B. C. Stone**	***Megacarpa***	**Appelhans MA679 (BISH, GOET, PTBG, USA)**	**Kauaʻi**
*Melicope knudsenii* (Hillebr.) T. G. Hartley & B. C. Stone	*Apocarpa*	Appelhans MA629 (silica sample only, ORPF)	Maui
***M. knudsenii*** **(Hillebr.) T. G. Hartley & B. C. Stone**	***Apocarpa***	**Oppenheimer H41610 (BISH)**	**Maui**
*M. knudsenii* (Hillebr.) T. G. Hartley & B. C. Stone	*Apocarpa*	Wood KW17119 (PTBG)	Kauaʻi
*Melicope lydgatei* (Hillebr.) T. G. Hartley & B. C. Stone	*Megacarpa*	Ching s.n. (silica sample only)	Oʻahu
***Melicope makahae*** **(B. C. Stone) T. G. Hartley & B. C. Stone**	***Apocarpa***	**Takahama s.n. (silica sample only)**	**Oʻahu**
*M. makahae* (B. C. Stone) T. G. Hartley & B. C. Stone (cf.)	*Apocarpa*	Appelhans MA609 (GOET, PTBG)	Oʻahu
*Melicope molokaiensis* (Hillebr.) T. G. Hartley & B. C. Stone	*Megacarpa*	Appelhans MA635 (BISH, GOET, PTBG)	Maui
*M. molokaiensis* (Hillebr.) T. G. Hartley & B. C. Stone	*Megacarpa*	Appelhans MA643 (BISH, GOET, PTBG, USA)	Maui
***M. molokaiensis*** **(Hillebr.) T. G. Hartley & B. C. Stone**	***Megacarpa***	**Oppenheimer s.n. (silica sample only)**	**Maui**
*Melicope mucronulata* (H. St. John) T. G. Hartley & B. C. Stone	*Apocarpa*	Appelhans MA630 (silica sample only, ORPF)	Maui
*Melicope munroi* (St. John) T. G. Hartley & B. C. Stone	*Megacarpa*	Oppenheimer s.n. (silica sample only)	Lanaʻi
*Melicope oahuensis* (H. Lév.) T. G. Hartley & B. C. Stone	*Cubicarpa*	Appelhans MA610 (BISH, GOET, PTBG, USA)	Oʻahu
M. oahuensis (H. Lév.) T. G. Hartley & B. C. Stone	*Cubicarpa*	Ching s.n. (silica sample only)	Oʻahu
***Melicope oppenheimeri*** **K. R. Wood, Appelhans & W. L. Wagner**	***Megacarpa***	**Wood KW7419 (PTBG)**	**Maui**
*M. oppenheimeri* K. R. Wood, Appelhans & W. L. Wagner	*Megacarpa*	Wood KW7408 (PTBG)	Maui
*Melicope orbicularis* (Hillebr.) T. G. Hartley & B. C. Stone	*Megacarpa*	Appelhans MA656 (BISH, GOET, PTBG, USA)	Maui
*M. orbicularis* (Hillebr.) T. G. Hartley & B. C. Stone	*Megacarpa*	Appelhans MA659 (GOET, PTBG)	Maui
*Melicope ovalis* (St. John) T. G. Hartley & B. C. Stone	*Cubicarpa*	Wood KW13724 (PTBG)	Maui
***Melicope ovata* (H. St. John & E. P. Hume) T. G. Hartley & B. C. Stone**	***Apocarpa***	**Appelhans MA662 (GOET, PTBG, USA)**	**Kauaʻi**
*M. ovata* (H. St. John & E. P. Hume) T. G. Hartley & B. C. Stone	*Apocarpa*	Appelhans MA684 (BISH, GOET)	Kauaʻi
M. ovata* (H. St. John & E. P. Hume) T. G. Hartley & B. C. Stone	*Apocarpa*	Appelhans MA663 (BISH, GOET, PTBG, USA)	Kauaʻi
M. ovata* (H. St. John & E. P. Hume) T. G. Hartley & B. C. Stone	*Apocarpa*	Wood KW17082 (PTBG)	Kauaʻi
M. ovata* (H. St. John & E. P. Hume) T. G. Hartley & B. C. Stone	*Apocarpa*	Wood KW16762 (PTBG)	Kauaʻi
*Melicope pallida* (Hillebr.) T. G. Hartley & B. C. Stone	*Apocarpa*	Appelhans MA689 (silica sample only)	Kauaʻi
*M. pallida* (Hillebr.) T. G. Hartley & B. C. Stone	*Apocarpa*	Wood KW16789 (PTBG)	Kauaʻi
*M. pallida* (Hillebr.) T. G. Hartley & B. C. Stone	*Apocarpa*	Wood KW15571 (PTBG)	Kauaʻi
***Melicope paniculata* (H. St. John) T. G. Hartley & B. C. Stone**	***Cubicarpa***	**Perlman 19387 (PTBG) = Appelhans MA660 (silica sample)**	**Kauaʻi**
*M. paniculata* (H. St. John) T. G. Hartley & B. C. Stone	*Cubicarpa*	Wood KW16155 (PTBG)	Kauaʻi
*Melicope peduncularis* (H. Lév.) T. G. Hartley & B. C. Stone	*Cubicarpa*	Appelhans MA652 (BISH, GOET, PTBG, USA)	Maui
*M. peduncularis* (H. Lév.) T. G. Hartley & B. C. Stone	*Cubicarpa*	Appelhans MA653 (BISH, GOET, PTBG, USA)	Maui
***Melicope pseudoanisata*** **(Rock) T. G. Hartley & B. C. Stone**	***Megacarpa***	**Appelhans MA632 (silica sample only, ORPF)**	**Maui**
*M. pseudoanisata* (Rock) T. G. Hartley & B. C. Stone	*Megacarpa*	Appelhans MA636 (silica sample only)	Maui
*M. pseudoanisata* (Rock) T. G. Hartley & B. C. Stone	*Megacarpa*	Appelhans MA642 (GOET, PTBG, USA)	Maui
*Melicope puberula* (H. St. John) T. G. Hartley & B. C. Stone	*Megacarpa*	Appelhans MA680 (GOET, PTBG, USA)	Kauaʻi
*M. puberula* (H. St. John) T. G. Hartley & B. C. Stone	*Megacarpa*	Wood KW16058 (PTBG)	Kauaʻi
*Melicope radiata* (H. St. John) T. G. Hartley & B. C. Stone	*Megacarpa*	Appelhans MA696	Hawaiʻi
***Melicope rostrata*** **(Hillebr.) Appelhans, K. R. Wood & W. L. Wagner**	***Platydesma***	**Appelhans MA683 (BISH, GOET)**	**Kauaʻi**
***Melicope rotundifolia*** **(A. Gray) T. G. Hartley & B. C. Stone**	***Megacarpa***	**Ching s.n. (silica sample only)**	**Oʻahu**
*Melicope sandwicensis* (Hook. & Arn.) T. G. Hartley & B. C. Stone	*Apocarpa*	Ching s.n. (silica sample only)	Oʻahu
*Melicope sessilis* (H. Lév.) T. G. Hartley & B. C. Stone	*Megacarpa*	Appelhans MA644 (BISH, GOET, PTBG, USA)	Maui
*Melicope* sp. (Rock) T. G. Hartley & B. C. Stone	*Megacarpa*	Wood KW17111 (PTBG)	Kauaʻi
*Melicope* sp. (Rock) T. G. Hartley & B. C. Stone	*Megacarpa*	Wood KW15733 (PTBG)	Kauaʻi
*Melicope spathulata* A. Gray	*Platydesma*	Appelhans MA697	Hawaiʻi
*M. spathulata* A. Gray	*Platydesma*	Wood KW16743 (PTBG)	Kauaʻi
*M. spathulata* A. Gray	*Platydesma*	Wood KW16836 (PTBG)	Kauaʻi
*Melicope stonei* K. R. Wood, Appelhans & W. L. Wagner	*Apocarpa*	Appelhans MA691	Kauaʻi
*M. stonei* K. R. Wood, Appelhans & W. L. Wagner	*Apocarpa*	Wood KW16727 (PTBG)	Kauaʻi
*Melicope volcanica* (A. Gray) T. G. Hartley & B. C. Stone (cf.)	*Megacarpa*	Oppenheimer s.n. (silica sample only)	Lānaʻi
*Melicope waialealae* (Wawra) T. G. Hartley `& B. C. Stone	*Pelea*	Wood KW16015 (PTBG)	Kauaʻi
**Outgroup**			
*Melicope aneura* (Lauterb.) T. G. Hartley		Appelhans MA418 (LAE, USA)	Papua New Guinea
*Melicope durifolia* (K. Schum.) T. G. Hartley		Appelhans MA455 (LAE, USA)	Papua New Guinea
*Melicope polyadenia* Merr. & L. M. Perry		Appelhans MA438 (LAE, USA)	Papua New Guinea
*Melicope triphylla* Merr.		Appelhans MA394 (GOET)	cultivated Hortus Botanicus Leiden
*Melicope brassii* T. G. Hartley		Appelhans MA436 (LAE, USA)	Papua New Guinea
*M. durifolia* (K. Schum.) T. G. Hartley		Appelhans MA465 (LAE, USA)	Papua New Guinea

Asterisk marks morphologically deviating specimens. Samples in bold were used in parameter optimization. ORPF, cultivated at Olinda Rare Plant Facility.

### RAD Library Preparation

DNA was extracted from silica-dried material using the Qiagen DNeasy Plant Mini Kit^®^ (Qiagen, Hilden, Germany) as per the manufacturer’s instructions with incubation in lysis buffer elongated to 2h. DNA concentration was measured using the Qubit^®^ fluorometer and the Qubit^®^ dsDNA BR Assay Kit (Thermo Fisher Scientific, Darmstadt, Germany) and adjusted to 30 ng/µL. Floragenex Inc. (Portland, Oregon, USA) generated RAD libraries using the restriction enzyme *Sfb*I. With a method following Baird et al. (2008) being employed, including the use of sample-specific barcodes, the samples were sequenced on an Illumina^®^ GAIIx platform to produce 100-bp single-end reads.

### RAD Locus Assembly

Quality of raw reads was checked using FastQC (Andrews, 2010). The program *ipyrad* v.0.7.21 was used to demultiplex raw reads allowing a mismatch of 1 bp. Raw reads were trimmed using cutadapt v.1.9.1 (Martin, 2011) as implemented in *ipyrad* by removing adapter sequences, trimming bases with Phred scores <30 and removing reads shorter than 35 bp after trimming. Trimmed reads were assembled *de novo* using the *ipyrad* pipeline. The software attempts to evaluate orthology by scoring alignments of reads or sequences, as opposed to assessing purely sequence identity (Eaton, 2014). The alignment score is the user-determined clustering threshold to be met. To reduce the risk of introducing assembly error to our dataset, we performed a modified clustering optimization approach (Paris et al., 2017). We iterated over core clustering parameters and plotted assembly matrices (cluster depth, heterozygosity, number of putatively paralogous loci, number of single-nucleotide polymorphisms (SNPs)) to identify parameters introducing excessive assembly errors (Paetzold et al., unpublished results; Paris et al., 2017). In addition, we optimized the clustering of reads within each individual sample and the clustering of consensus sequences across loci separately, reasoning that the divergence found within each individual genome might be significantly different from the ca. 10 Myr of divergence (Appelhans et al., 2018a) within the lineage as a whole. Thus, the assembly was generated using a clustering threshold of 95 for in-sample clustering and 90 for between-sample clustering. The final filtering of loci was performed for values 10, 32, 50, 67, and 85 as the minimum numbers of samples per locus.

### Phylogenetic Inference and Quartet Sampling

Phylogenetic inference was performed on all resulting alignments using maximum likelihood (ML) and Bayesian inference (BI). As individual loci are very short and may comprise a high fraction of missing data, a partitioned analysis is neither computationally feasible nor expected to produce reliable results. Thus, all datasets were analyzed solely concatenated. ML was performed using ExaML v3.0.2 (Kozlov et al., 2015) using the new rapid hill-climbing algorithm, a random number seed, the gamma model of rate heterogeneity, and the median for discrete approximation of rate heterogeneity. For datasets containing minimum numbers of 10, 32, and 50 samples, the memory saving option for gappy alignments was activated (-S). Parsimony starting trees were generated using RAxML v8.2.4 (Stamatakis, 2014). RAxML was also used to generate 100 bootstrap replicate alignments and their corresponding parsimony starting trees. ExaML searches were run on every replicate alignment with the above-mentioned settings.

BI was performed using ExaBayes v 1.5 (Aberer et al., 2014). Four independent runs were carried out with a convergence stopping criterion (split frequencies average <5% in three subsequent generations) and for a minimum of 100,000 generations sampling every 100th generation under the GTR+I+G model. Majority rule consensus trees were drawn on topologies of all four runs combined after the first 25% was discarded as burn-in.

Analysis of large-scale, concatenated datasets can result in erroneous relationships with high bootstrap support because of a failure to model the effects of ILS (Gadagkar et al., 2005; Kubatko and Degnan, 2007; Seo, 2008). These effects can be driven by only a few loci (Shen et al., 2017) and especially pertain to short branches (Kumar et al., 2012). On the other hand, a simulation study has shown that concatenated analysis of datasets containing loci with anomalous gene trees will more likely result in unresolved species tree topologies, rather than highly supported false ones (Huang and Knowles, 2009).

Methods implementing the multispecies coalescent (MSC) model explicitly incorporate gene tree conflict into species tree inference and are thus more robust to ILS than concatenation approaches (Kubatko and Degnan, 2007) but are often intractable for large datasets (Liu et al., 2015). Summary methods of species tree inference under the MSC, for example, ASTRAL (Mirarab et al., 2014) or NJst (Liu and Yu, 2011), are based on the analysis of individual gene trees and have become popular due to their comparative speed and accuracy. However, the limited information content of individual RAD loci often limits their application for gene tree inference, which may negatively impact species tree estimation (Salichos and Rokas, 2013; Mirarab et al., 2016). Alternatively, site-based methods avoid estimation of gene trees, instead using SNP data directly, and so are expected to be well suited to short, low-variability loci (Molloy and Warnow, 2018). We employed the SVDQuartets method, which infers quartet trees from SNPs using phylogenetic invariant patterns under the coalescent model and then infers the species tree by quartet joining of the subtrees using algebraic statistics (Chifman and Kubatko, 2014). We converted the SNP datasets into nexus format using the Ruby script *convert_vcf_to_nexus.rb* (Matschiner, 2018). The SVDQuartets analysis was computed as implemented in the software PAUP*4.0a (Swofford, 2002; Swofford, 2018). We analyzed 250,000 randomly selected quartets and assessed statistical support using 100 nonparametric bootstrap support replicates. For ambiguous positions in the SNP matrix, we chose the “Distribute” option, as these positions represent heterozygous sites.

To estimate the robustness of resolved relationships, we employed the Quartet Sampling method, which aims to measure branch support in large sparse alignments (Pease et al., 2018). As each internal branch divides all samples within a phylogeny into four non-overlapping subsets, the method randomly samples one taxon per subset to produce a quartet phylogeny. The topology of each quartet is either concordant with the tree topology or discordant. Discord is measured and quantified to produce four metrics—quartet concordance (QC), quartet differential (QD), quartet informativeness (QI), and quartet fidelity (QF)—allowing effective assessment of branch-related (QC, QD, and QI) and taxon-related (QF) discordance in the dataset (Pease et al., 2018). The method is implemented in the python script *quartet_sampling.py* (https://www.github.com/fephyfofum/quartetsampling). We performed Quartet Sampling on all datasets and the respectively resolved topologies using 500 replicates per branch with a minimum required overlap of 300,000 bp in the min10, min32, min50, and min67 concatenated datasets. The minimum overlap was lowered to 140,000 bp in the min85 concatenated dataset, as otherwise five samples would have been excluded from the analysis.

### Test for Introgression

**Figure 1:**
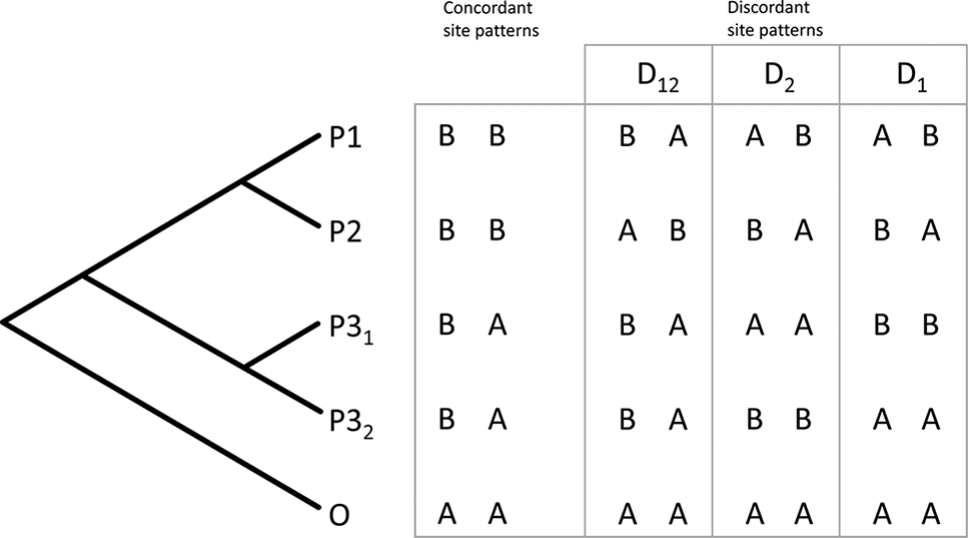
The principle of five-taxon *D*-statistics test. Biallelic site patterns are quantified, which support or contradict the underlying symmetric phylogeny. Asymmetry of discordant site patterns is quantified to calculate three separate *D*-statistics characterizing introgression from the P3_1_ taxon (D_1_), the P3_2_ taxon (*D*_2_), or their common ancestor (*D*_12_) into the taxa designated P_1_ and P_2_ (Eaton and Ree, 2013).

The *D*-statistics (Durand et al., 2011) is a site-based test for introgression. In a four-taxon topology (((P1, P2), P3), O), a derived allele in the P3 lineage is expected to occur also in either P1 or P2 with equal frequency, giving rise to either an ABBA or BABA discordant site pattern (Durand et al., 2011). A statistically significant imbalance in these site pattern frequencies provides evidence of introgression, while equal frequencies are associated with neutral processes like ILS. Unfortunately, this test is not well suited for deeper evolutionary timescales, where the P3 lineage has diverged into multiple sub-lineages, and it also does not allow inference of direction of introgression. Partitioned *D*-statistics is a system of multiple four-taxon *D*-statistics in a symmetric, five-taxon phylogeny with the ingroup taxa forming two pairs (P1, P2) and (P3_1_, P3_2_) and an outgroup taxon (O) (**Figure 1**) (Eaton and Ree, 2013). The partitioned *D*-statistics identifies sites, in which either or both of the P3 lineages share a derived allele with either P1 or P2, but not both (**Figure 1**) (Eaton and Ree, 2013).

We used partitioned *D*-statistics to infer whether discordant relationships inferred between major clades (see below) are caused by ILS or introgression. We defined entire clades as lineages and tested all combinations obeying the symmetric topology.

## Results

### Raw Data and Assembly

Illumina Sequencing yielded an average of 10,439,082 reads per sample (342,914–34,663,109). After quality trimming, an average of 10,327,562 reads per sample (271,257–34,542,777) were left. The assembled dataset contained a total of 786,169 clusters prior to filtering by sample coverage. Filtering reduced the number of loci by over 90% (**Table 2**). The final datasets contained between 7,266 (min85) and 59,041 (min10) loci. The number of variable sites (SNPs) ranged from 529,045 (min10) to 82,760 (min85) (**Table 2**).

**Table 2:** Differences between the number of loci, their concatenated length, and the number of SNPs resulting from filtering by minimum samples per locus (10, 32, 50, 67, and 85).

	Total	min10	min32	min50	min67	min85
Number of loci	786,169	59,041	36,622	30,801	23,401	7,266
Concatenated length (bp)	NA	4,800,367	2,986,760	2,506,242	1,892,473	584,086
Number of SNPs	NA	529,045	385,871	332,935	256,276	82,760

### Phylogenetic Inference

**Figure 2:**
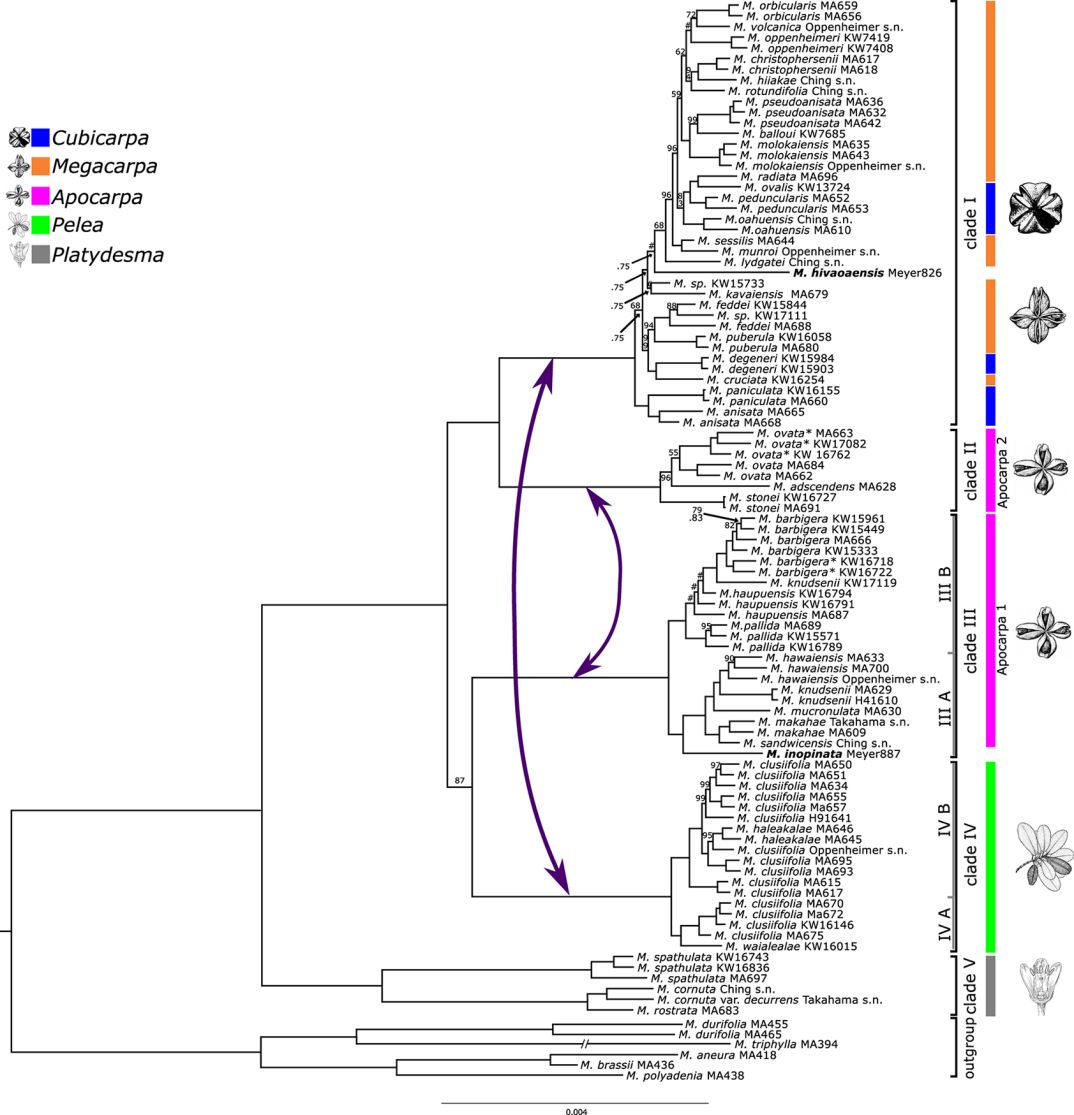
Phylogeny of Hawaiian *Melicope* based on the concatenated min32 dataset. Bayesian posterior probability (PP) values are indicated above branches, and maximum likelihood (ML) nonparametric bootstrap support (ML-NBS) below branches. Support values are not shown for maximally supported clades (1.00pp/100BS). A hashtag (#) represents incongruent species relationships between Bayesian and ML analyses. Clade colors and line drawings correspond to morphologically limited Stone’s sections. Bold samples represent Marquesan species. Asterisks mark specimens differing morphologically from the typical representatives of these species. Purple arrows mark putative introgression events.

All five final datasets were used for phylogenetic inference in concatenated BI, ML, and SVD Quartets analyses. Statistical support for inferred relationships was assessed using posterior probabilities (PPs), nonparametric bootstrap (NBS) (ML-NBS and SVD-NBS), and Quartet Sampling. Analyses of the five datasets resulted in mostly congruent relationships, with few exceptions (see below). NBS and PP values are very high across the trees. QI values are high for all nodes (>0.9), and QF scores are average between 0.83 and 0.88 across datasets. **Figure 2** shows the result of phylogenetic inference in the concatenated min32 dataset.

**Figure 3:**
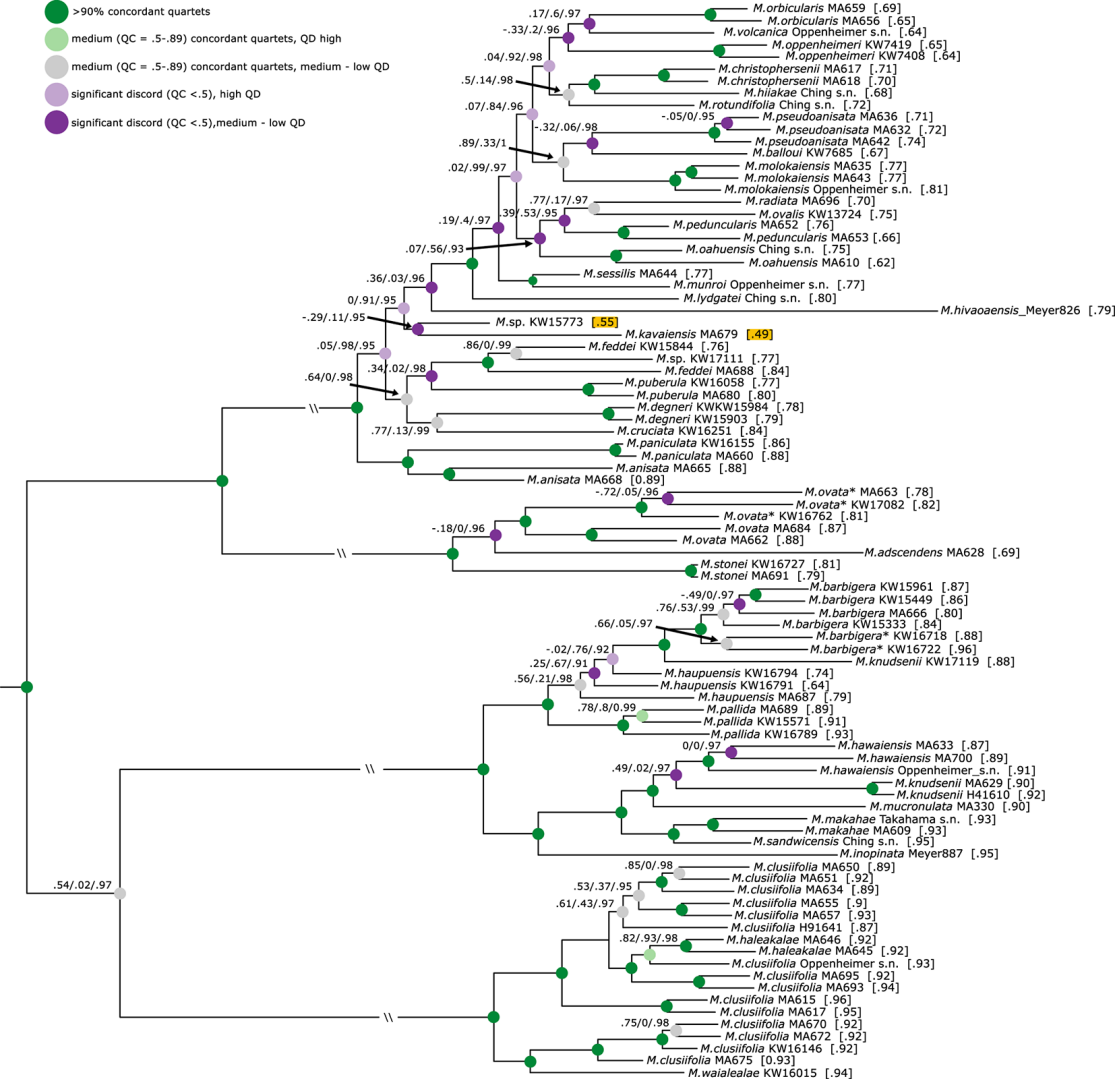
Phylogeny of Hawaiian *Melicope* based on the min32 dataset. Quartet Sampling results (quartet concordance (QC)/quartet differential (QD)/quartet informativeness (QI)) are indicated on branches, and quartet fidelity (QF) values behind samples. Nodes are colored according to QC and QD values. Results are not shown for branches with QC > 0.9. The lowest QF values are highlighted. Outgroup specimens are removed for graphical purposes. All outgroup relationships receive maximum QC values (1/-/1).

Hawaiian *Melicope* are divided into five main clades corresponding to those previously resolved by Appelhans et al. (2014b). These five clades are fully supported by all statistical methods. The former genus *Platydesma* represents the earliest diverging lineage (clade V; **Figure 2**). Clade IV corresponds to Stone’s section *Pelea*, characterized by whorled leaves. The remaining Stone sections appear to be non-monophyletic. Species ascribed to Stone’s section *Apocarpa* are resolved as two independent lineages (Clades II and III). Clade I comprises all species of Stone’s sections *Cubicarpa* and *Megacarpa* intermingled (**Figure 2**). Relationships of clade III were resolved incongruently between datasets and analyses. BI and ML analyses resolved clade III as sister to clade IV, and the resulting monophyletic lineage again in a sister–group relationship to clades I + II with maximum PP and high ML-NBS support in four of the datasets (min10, min32, min50, and min85), yet with some discord detected by Quartet Sampling (**Figures 2** and **3**, **Supplemental Figures 1**, **2**, and **4**). The concatenated min67 dataset resolves clade III as sister to clades I + II, and clade IV as sister to clades I + II + III (**Supplemental Figure 3**) with medium statistical support. Coalescent-based SVDQuartets analysis of SNP datasets resolved a third alternative topology. Here, clade II is resolved as sister to clade III, and the resulting lineage is sister to clades I + IV. This topology receives medium-to-low SVD-NBS support across all SNP datasets, as well as medium-to-high negative QC values, indicating substantial counter-support for this relationship (**Supplemental Figures 5–9**). The relationship of clade III is highly discordant over quartet replicates (**Supplemental Figure 3**). Across all datasets, the discord detected by Quartet Sampling for the ancestral branch is skewed favoring one of the tested alternative quartet topologies (QD; **Figure 3**, **Supplemental Figures 1–9**).

The remaining relationships within individual clades are fully resolved, improving resolution to the species and intraspecies levels (**Figure 2**). The majority of all Hawaiian *Melicope* are resolved in clade I, and relationships among species show many nodes with notable discord and very short branches (**Figure 2**). Most of the nodes show low QC and medium-to-low QD values (**Figure 3**). Three samples show incongruent relationships between datasets. This pertains to the Marquesan *Melicope hivaoaensis*, which is resolved in clade I as either sister to the remaining species (**Supplemental Figures 3–9**) within the clade or diverging prior to *Melicope lydgatei* (**Figures 2** and **3**, **Supplemental Figures 1** and **2**) as well as to *M. kavaiensis* and *Melicope* sp. KW15773 (**Figure 3**, **Supplemental Figures 1–9**). In all datasets, QC values show high discord or even counter-support for the placement of these three specimens. However, while QD and QF values are high for *M. hivaoaensis*, for both *M. kavaiensis* and *Melicope* sp. KW15773, QD values are low and QF scores are below average (0.47–0.6 for *M. kavaiensis*) (**Figure 3**, **Supplemental Figures 1–9**). The remaining relationships in clade I are congruent among all concatenation-based analyses. Site-specific coalescence analysis, however, resolved largely incongruent relationships for taxa in this clade, especially pertaining to the most recent divergences. The inferred relationships receive medium-to-very-low SVD-NBS values and show a high amount of discord in Quartet Sampling (**Supplemental Figures 5–9**).

Clades III and IV are subdivided into two subclades each. Most species sampled with multiple accessions are resolved as monophyletic with high support and no discord detected in Quartet Sampling. Exceptions are *Melicope clusiifolia*, *Melicope haupuensis*, *Melicope knudsenii*, and *Melicope feddei*. *M. clusiifolia* is resolved paraphyletic with respect to *Melicope haleakalae*, which is nested within clade IVB with high-to-maximum support. Specimens of *M. haupuensis* are resolved as polyphyletic within clade IIIB. The relationships among the three sampled taxa are not resolved consistently across datasets and poorly supported. Quartet Sampling reveals a high level of discord and below-average QF scores (**Figure 3**, **Supplemental Figures 1–9**). *M. knudsenii* is also resolved as polyphyletic with two Maui specimens (MA629 and H41610) monophyletic in clade IIIA, while the third sample from Kauaʻi (KW17119) is resolved as sister to *M. barbigera* in clade IIIB (**Figure 2**). Either relationship is virtually uncontested (**Figures 2** and **3**, **Supplemental Figures 1–9**). *M. feddei* is paraphyletic with respect to one of the Kauaʻi *M. wawraeana*-like specimens (KW17111). The three individuals form a fully supported, monophyletic unit (**Figures 2** and **3**, **Supplemental Figures 1–9**).

None of the three species complexes (*M. elliptica*, *M. kavaiensis*, and *M. volcanica* complexes) are resolved as monophyletic. Species of both the *M. kavaiensis* and *M. volcanica* complexes are resolved in clade I (**Figure 2**) in proximity to each other, but not sister to each other. Species of the *M. elliptica* complex are resolved in different subclades of clade III (**Figure 2**). Both *M. barbigera* and *M. ovata* were resolved as monophyletic, and the morphologically divergent specimens (**Table 1**, asterisk) are resolved as sister clades to the samples with the typical morphology of the respective species with high support (**Figure 2**).

The species from the Marquesas Islands are deeply nested within the Hawaiian clade.*Melicope inopinata* is resolved in clade III as sister to the rest of subclade IIIA. *M. hivaoaensis* represents a group of six morphologically similar species that form a highly supported monophyletic clade (Appelhans et al., 2014b; Appelhans et al., 2018a) and is nested within clade I here (**Figure 2**).

### Test for Introgression

The min32 dataset was used for the ABBA-BABA test, since it produced the highest number of fully supported nodes. The tree topology in **Figure 2** was chosen to represent the species tree topology, as it was recovered by the majority of analyses. The *D*-statistics was only used to test the incongruent position of clade III, as for incongruent species within clade I, the sampling of the respective populations is not sufficient to draw reliable conclusions. Samples within clades were pooled, and SNP frequencies were used for *D*-statistic calculations (Durand et al., 2011). All possible relationships complying with the *D*-statistic assumptions were tested. A total of 24,673 loci covered at least one-third of all samples per clade and, thus, contributed to the test results. **Table 3** summarizes the tested topologies and inferred partitioned *D*-statistics. When clades III and IV are tested as donors for introgressed loci, values for *D*_12_ are small and not significant (*Z*_12_ < 2.55), while values for *D*_1_ and *D*_2_ are significant, respectively. For tests with either of clade I or II designated as P3 lineages, *D*_12_, and *D*_1_ and *D*_2_, are all significant (**Table 3**). For all tested configurations, the dataset exhibits more than 3,000 discordant site patterns (**Table 3**).

**Table 3:** Partitioned *D*-statistics for introgression involving clades I–IV.

((P1, P2), (P3_1_, P3_2_), O)	*D*_12_	Z_12_	*n* ABAAA	*n* BABBA
((I, II), (III, IV), V&O)	0.020	0.95	809.84	778.1
((I, II), (IV, III), V&O)	−0.020	0.96	778.1	809.84
((IV, III), (I, II), V&O)	0.066	3.28	1,273.58	1,115.49
((IV, III), (II, I), V&O)	−0.066	3.29	1,273.58	1,115.5
				
((P1, P2), (P3_1_, P3_2_), O)	*D*_1_	Z_1_	*n* ABBAA	*n* BABAA
((I, II), (III, IV), V&O)	0.276	8.48	261.01	437.67
((I, II), (IV, III), V&O)	−0.276	8.17	437.67	261.01
((IV, III), (I, II), V&O)	−0.242	7.07	403.07	222.05
((IV, III), (II, I), V&O)	0.290	7.61	271.16	444.45
				
((P1, P2), (P3_1_, P3_2_), O)	*D*_2_	Z_2_	*n* ABABA	*n* BAABA
((I, II), (III, IV), V&O)	−0.253	7.08	505.48	286.73
((I, II), (IV, III), V&O)	0.253	7.05	286.73	505.48
((IV, III), (I, II), V&O)	0.290	7.57	271.16	444.45
((IV, III), (II, I), V&O)	−0.242	7.24	403.06	222.05

Z scores ≥2.55 represent a significant value for D_x_. The respective numbers of concordant and discordant site patterns are listed. Clade numbers refer to those in **Figure 2**, and the group they are assigned to in the partitioned D-statistics test is indicated (compare **Figure 1**). O, outgroup.

## Discussion

### Phylogeny and Introgression

Analysis of *ipyrad* assemblies consistently resolved five major clades within Hawaiian *Melicope* (**Figure 2**). However, the relationships of clade III were incongruent among the five datasets and analysis methods (**Figure 2**, **Supplemental Figures 1–9**). Incongruence between datasets may be caused by one of three factors: noise, ILS, or non-tree-like evolution. As noise is expected to impact small datasets and deep nodes most severely (Misof et al., 2014), it is unlikely a sufficient cause of the incongruence observed here, since our RAD-seq alignments are substantial in size (**Table 1**) and the remaining deep nodes are not affected.

The QD values of the branch illustrate that one of the discordant topologies is inferred significantly more often (0.0–0.4; **Figure 3**, **Supplemental Figures 1–9**), which indicates non-tree-like evolution as the cause for the discord. Thus, we used the partitioned *D*-statistics to test for signals of ancient introgression between clades I through IV with all clades tested as putative donor (P3) lineages. In all cases, values for *D*_1_ and *D*_2_ were each significant, yet values for *D*_12_ were only significant when clades I and II were defined as P3 (**Table 3**). Positive values of *D*_1_ represent introgression between P2 and P3_1_, while negative values indicate introgression between P1 and P3_1_, and values for *D*_2_ represent events analogous for P3_2_ and P2 (Eaton and Ree, 2013; Pease and Hahn, 2015). The significant values for *D*_1_ and *D*_2_ indicate introgression between the respective ancestors of clades I and IV as well as between respective ancestors of clades II and III. Significant values for *D*_12_ represent shared ancestral alleles from the clade I + II progenitor introduced into the respective ancestor of clades III and IV (**Figure 2**, **Table 3**). All taxa in clades II and III have apocarpous fruits, while all taxa in clades I and IV have syncarpous fruits (Stone et al., 1999), providing a morphological connection between either of the two pairs, which might be linked to introgressed information. However, we interpret these result cautiously, as *D*-statistic results are sensitive to confounding signals from multiple introgressive events due to phylogenetic non-independence of tests (Eaton et al., 2015).

The origin of the Hawaiian *Melicope* lineage predates the rise of the current high islands (Appelhans et al., 2018a). Thus, the inferred introgressive events are associated with a time when the ancestral species were still relegated either to refugial areas on small, low islands or shortly after they colonized the young island of Kauaʻi. The time frame under consideration presents a “bottleneck” scenario, where the ancestral lineages were likely in close spatial proximity. Additionally, increased volcanic activity of the Hawaiian hot spot coincided with the rise of Kaua'i (Price and Clague, 2002). This volcanic activity could have produced lava flows, earthquakes, tsunamis, and other catastrophic events, which may have additionally promoted hybridization (Stuessy et al., 2014). The ancestral hybridization events may even have promoted subsequent adaptive radiation on the islands (Kagawa and Takimoto, 2018). Estimation of divergence times in Hawaiian *Melicope* will be needed to infer the time frame for hybridization events in ancestral lineages. While there is strong evidence for ancient hybridization events within Hawaiian *Melicope*, the nature of *de novo* RAD-seq data currently limits our analytic methods. Further information may be obtained through gene tree-based approaches applied to target capture or whole genome-sequencing data (Meng and Kubatko, 2009) or by examining SNP-based patterns, as they vary spatially along a reference genome (Martin et al., 2013).

Bootstrap and PP support values were generally high across trees inferred from different datasets but generally increased with dataset size. Lenient filtering in RAD-seq data is often practiced, as there is a correlation between the size of a data matrix and resolution and support of relationships (Wagner et al., 2013; Hodel et al., 2017). RAD locus dropout is expected to increase with increasing divergence times, as enzyme cut sites will be lost or gained through mutation (Cariou et al., 2013). Loci with a small amount of missing data are therefore expected to represent the conserved spectrum of genomic sites and, thus, provide a limited capacity of resolution. On the other hand, sparse loci are expected to iincrease resolution of relationships despite also introducing noise, as they are assumed to represent the more rapidly evolving genomic fractions (Cariou et al., 2013; Wagner et al., 2013; Eaton et al., 2015). However, including all loci is not advisable either, as there seems to be a point at which inclusion of increasingly more sparse loci might start to decrease support. At this point, noise, due to missing data introduced by the inclusion of more sparse loci, will overpower the informative value these loci provide. However, the Quartet Sampling method seems an adequate approach to evaluating the reliability of the dataset, as the QC value showed the same trend in all datasets regardless of size and offer the QI score to assess the amount and impact of missing data.

We detected some discord between relationships resolved by concatenation and site-specific coalescence-based methods (**Figure 2**, **Supplemental Figures 1–9**). The evaluation of the performance of different species-tree inference methods is a matter of ongoing research, especially with regard to genomic datasets. Concatenation-based ML inference can be statistically inconsistent under some conditions in the MSC, that is, ILS causing gene trees to differ from the true species tree (Kubatko and Degnan, 2007). However, the limits of the concatenated approach are poorly understood (Molloy and Warnow, 2018), and the performance of concatenated Bayesian analysis has yet to be formally assessed. Some simulation studies show that concatenated RAD-seq data are robust to gene tree/species tree discord when inferring relationships among taxa (Rivers et al., 2016). In addition, concatenated approaches potentially offer hidden support as a feature overriding gene tree/species conflict (Gatesy and Springer, 2014; Rivers et al., 2016), although hidden support has not been addressed in plant phylogenomic research yet. Coalescence-based methods are statistically consistent under the MSC. Bayesian co-estimation of gene trees and the species tree under the MSC is currently considered the most effective approach, yet computationally very demanding and thus less applicable to large datasets. Hence, summary and site-specific MSC methods have become popular, and several algorithms implementing the concepts do exist (Liu et al., 2015). However, the assessment of the performance of these methods under empirical and simulated conditions is still a matter of active research. For example, gene tree methods have proven to be statistically inconsistent if the cause of gene tree discord is horizontal gene transfer, instead of ILS (Solís-Lemus et al., 2016; Fernández-Mazuecos et al., 2018). Several recent simulation studies compared the accuracy of multiple summary and site-based coalescent methods, including SVDQuartets, as well as concatenated ML under varying levels of ILS and gene tree estimation error (GTEE). Concatenated ML was at least competitive with MSC methods under most conditions and outperformed SVDQuartets under all tested conditions, including high GTEE (Chou et al., 2015; Mirarab et al., 2016; Molloy and Warnow, 2018). The latter would be expected in RAD-seq datasets and should also be present herein.

With respect to species relationships inferred for Hawaiian *Melicope* and considering the observed lower accuracy of SVDQuartets compared with concatenation-based approaches under conditions typically characterizing RAD datasets, we suggest that the results from concatenated BI and ML are probably more accurate than those based on SVDQuartets and will be discussed below. However, we do stress that none of the approaches have proven to be statistically consistent under conditions observed herein, that is, ILS, GTEE, and horizontal gene transfer (**Figure 2**).

### Taxonomic Implications

The former small genus *Platydesma* and Stone’s section *Pelea* are each monophyletic (**Figure 2**), while the three remaining sections of Stone, comprising the majority of all Hawaiian *Melicope* species, are not. *Apocarpa* is divided into two lineages with the majority of species resolved in *Apocarpa* 1 (**Figure 2**). The three species of the *Apocarpa* 2 clade share a number of morphological traits, though none of them is either exclusive or inclusive. All species of *Apocarpa* 2 occur in mesic forests only and, with the exception of *Melicope stonei*, share a sprawling, shrubby habit (Stone et al., 1999; Wood et al., 2017). Finally, in all *Apocarpa* 2 species, both endocarp and exocarp are glabrous and inflorescences are few-flowered, though both of these traits also appear outside of this group (Stone et al., 1999; Wood et al., 2017). In a previous analysis, apocarpous species were resolved in three different clades (Appelhans et al., 2014b), one of which, consisting of *M. elliptica* only, could not be sampled in this study. Further research will be necessary to identify morphological character combinations distinguishing these lineages. Stone’s sections *Cubicarpa* and *Megacarpa* are paraphyletic with respect to each other (**Figure 2**) with species of each resolved intermingled throughout the clade. The two groups differ by the degree of carpel connation, with carpels “connate from base up to 2/3 of their length” (Stone et al., 1999) characterizing *Megacarpa* and carpels “nearly to completely” connate (Stone et al., 1999) characterizing *Cubicarpa*. Carpel connation clearly represents a continuum and not two discrete units. As there is no pattern to the degree of carpel fusion apparent in clade I, the separation of these two of Stone’s sections seems artificial.

Interspecies relationships within clade I are less well supported than in the remaining clades, and Quartet Sampling reveals measurable discord at nearly every branch in the backbone of this clade. For many of the nodes with low QC values, QD values are high (**Figure 3**, **Supplemental Figures 1–9**), which characterizes ILS and corresponds to the shortness of these branches. On the other hand, many branches show low QD values, indicating widespread introgression between these lineages. Unfortunately, sampling herein is not sufficient to test individual relationships.

Of the 24 species represented by multiple accessions, 20 were resolved as monophyletic, while four species were either paraphyletic or polyphyletic. *M. clusiifolia* is the most widespread and morphologically diverse of all Hawaiian *Melicope* (Stone et al., 1999), and it is paraphyletic with both of the other species of Stone’s section *Pelea*, *M. haleakalae* and *Melicope waialealae* (clade IV, **Figure 2**). Several attempts have been made to subdivide *M. clusiifolia* into varying constellations of subspecies, varieties, and forms (St. John, 1944; Stone, 1969). In the most recent taxonomic treatment, Stone et al. (1999) synonymized all subdivisions of the species, arguing that the variable characters seem to represent a continuum rather than distinguishable, discrete units. However, the authors also issued the recommendation that the overall pattern of variability in *M. clusiifolia* should be studied in detail (Stone et al., 1999). *M. haleakalae* is characterized as differing from *M. clusiifolia*, mainly in its persistent sepals (Stone et al., 1999). Considering that *M. haleakalae* is nested deeply within *M. clusiifolia* (**Figure 2**, clade IV), the two might be regarded as conspecific and included in an overall evaluation of the complex. *M. waialealae* differs from *M. clusiifolia* mainly in leaf shape (Stone et al., 1999). However, since the leaf shape of *M. clusiifolia* is highly variable, *M. waialealae* might represent one end of a continuum across both taxa rather than one of two distinct states. On the other hand, these three species might represent a case of speciation in progress. In this case, the deep nesting, especially of *M. haleakalae*, within *M. clusiifolia*, would represent speciation following a progenitor-derivative scenario (Crawford, 2010). The widespread, morphologically variable *M. clusiifolia* would meet all criteria of the progenitor (p) species. The persistent petals in *M. haleakalae* and the leaf shape in *M. waialealae* would represent a variable, morphological feature in the parent being fixed in the respective derivative (d) species. Identification of a true p–d relationship is difficult and rare. However, several candidate species pairs do exist (Crawford, 2010). The p–d species pair *Layia glandulosa* (Hook.) Hook. & Arn. and *Layia discoidea* D. D. Keck (Asteraceae) show not only a shift in morphology between progenitor and derivative species but also geographic isolation due to a shift in habitat (Baldwin, 2005). This could be the same for *M. waialealae*, which is restricted to bogs, whereas the putative progenitor *M. clusiifolia* occurs in mesic to wet forests (Stone et al., 1999). Unfortunately, there are no data available regarding breeding system or pollinator communities in these species, creating potential barriers to gene flow. Detailed studies of morphological characters, gene flow, and abiotic habitat factors are necessary to determine whether these taxa are separate p–d species pairs or conspecific, as already indicated in previous studies (Appelhans et al., 2014b).

*M. knudsenii*, delimited by Stone et al. (1999) as the only species occurring on non-adjacent islands, was resolved as polyphyletic, with three samples resolved as two distinct lineages within clade III. Appelhans et al. (2014b) already showed that this taxon is polyphyletic, consisting of three taxa. One of these was recently described as *M. stonei* (Wood et al., 2017). Our results confirm the previously resolved pattern with the two specimens of *M. knudsenii* from Maui resolved as sister to *Melicope hawaiensis* and the specimen from Kauaʻi as sister to *M. barbigera* (clade III, **Figure 2**). We confirm that these specimens clearly represent different species. The Maui species will be resurrected under one of the names used in an earlier treatment by Stone (1969), wherein he adopted a narrower species concept than in the later classification (Stone et al., 1999), leaving *M. knudsenii* restricted to only populations on Kauaʻi.

The three specimens of *M. haupuensis* included in this study are resolved as paraphyletic. Moreover, they are the only species resolved with incongruent topologies of the individual samples associated with the different datasets (compare **Figure 2**, **Supplemental Figures 1–9**). Quartet Sampling shows strong discord for either of the inferred relationships with medium QD values (**Figure 3**, **Supplemental Figures 1–9**), indicating the possibility of introgressed sites. Moreover, QF scores for the three specimens are considerably lower than the average, indicating a rogue behavior (Aberer et al., 2013; Pease et al., 2018) of the three taxa. Additionally, several specimens in the field were observed presenting morphologically intermediate forms between *M. haupuensis* and *M. barbigera* (personal observation K.R. Wood). QD values for the latter are also low (**Figure 3**, **Supplemental Figures 1–9)**. Both the morphological intermediates and the incongruence associated with different datasets indicate potential hybridization between these species. However, conclusively identifying putative hybridization events would require sampling at the population level, including any morphological intermediates.

Multiple samples of *M. ovata* and *M. barbigera* were included in our analyses, representing both the typical morphology and a deviating morphotype. For either species, the morphologically deviating samples were resolved as the sister group to the samples with the typical habit. Variant morphotypes of *M. ovata* displayed a pubescent lower leaf surface, whereas leaves are typically glabrous in this species. *M. barbigera* usually has few-flowered inflorescences (Stone et al., 1999). In contrast, the variant morphotype has inflorescences with a considerably larger number of flowers. Genomic divergence is comparable with that of other species pairs within the lineage. Both groups might be another case of speciation in progress within Hawaiian *Melicope*. In both cases, detailed morphological studies will be necessary to investigate if the morphologically divergent populations of the two species should be recognized as separate taxa.

The two *M. wawraeana*-like specimens are resolved in clade I, but not closely related to each other. One specimen (KW17111) is nested within the two samples of *M. feddei* with high support (**Figures 2** and **3**). *M. wawraeana* is very similar to *M. feddei* and differs mainly in pedicel length (Stone et al., 1999). The present results suggest that some populations might be conspecific with *M. feddei*, while others (e.g., from the herein unsampled type location) are not. The relationships of the second *M. wawraeana*-like specimen (KW15733) are resolved incongruently among datasets, as are the relationships of the sampled specimen of *M. kavaiensis*. The two samples are resolved either as sister groups (**Figure 3**, **Supplemental Figures 1**, **4**, **5**, and **7–9**) or as consecutive sister clades within clade I (**Supplemental Figures 2**, **3**, and **6**). There is a substantial amount of discord in the dataset for either of the resolved relationships. QD values are low, indicating the possibility of introgression between these morphologically distinct species. Additionally, QF scores for either of the specimens are low corresponding to the rogue behavior of the samples.

The rogue behavior of the aforementioned samples (*M. kavaiensis*, *Melicope* sp. KW15733) might also be related to the incongruent placement of *M. hivaoaensis*, as the three taxa are inferred as closely related, regardless of the relation to the remainder of clade I. For this specimen, QC and QD values are low; however, QF is high (**Figure 3**, **Supplemental Figures 1–9**). *M. hivaoaensis* represents an adaptive radiation of five species endemic to the Marquesas Islands, whose predecessor colonized from the Hawaiian Islands (Appelhans et al., 2014a; Appelhans et al., 2018a). Successful island colonizations have been associated with recent hybridization or polyploidization events (Paetzold et al., 2018). There was no polyploidization event immediately prior to the colonization of the Hawaiian Islands itself (Paetzold et al., 2018), making a polyploidization event prior to the colonization of the Marquesas Islands unlikely. Chromosome counts for Marquesan species are not available for a conclusive answer. However, results herein indicate the presence of several hybridization events within the lineage. Thus, a hybridization event might have predated the colonization of the Marquesas Islands as well. As the incongruent position of *M. hivaoaensis* seems to correspond to the rogue behavior of *Melicope* sp. KW15733 and *M. kavaiensis*, the latter two might represent the parental lineages of the Marquesan *Melicope* radiation. A conclusive answer to the question is contingent on a thorough sampling of all concerned lineages as well as a prior revision of the *M. wawraeana* species concept.

We confirm previous results showing that Hawaiian *Melicope* colonized the Marquesas Islands twice independently, negating the hypothesis that the remote Hawaiian Islands constitute a dispersal sink (Harbaugh et al., 2009; Appelhans et al., 2014a; Appelhans et al., 2018a). The nesting of Marquesan species in different Hawaiian clades is corroborated by fruit morphology (Hartley, 2001), since *M. hivaoaensis* and its close relatives from the Marquesas Islands have syncarpous fruits as do the species in clade I, while *M. inopinata* has apocarpous fruits like the species in clade III.

The present study provides unprecedented insight into the relationships of Hawaiian *Melicope*. Several previous findings could be corroborated and firmly supported by genome-wide data, including the non-monophyly of most of Stone’s sections, which cannot be held up as delimited (Stone, 1969; Stone et al., 1999). The lineage is in need of a taxonomic revision. Understanding the relationships of Hawaiian *Melicope* would be enhanced by some formal recognition of the subclades with corresponding morphological features. However, the creation of novel formal subgroups within *Melicope* section *Pelea* must also include the extra-Hawaiian members of the section. The former genus *Platydesma* is the most distinctive group within *Melicope* sect. *Pelea* and should receive some level of formal recognition. *Apocarpa* species need to be split into two groups, one of which would include the Marquesan species *M. inopinata*. However, conclusive treatment of *Apocarpa* should be adjourned until an improved understanding of the separation within the *M. elliptica* complex is attained. Delimitations of species within the *Pelea* group, *M. barbigera*, *M. ovata*, and *M. haupuensis*, may need revision, but levels of hybridization should also be investigated as part of that process. *M. wawraeana* requires revision as well as a prerequisite to test the putative hybrid character of the Marquesan radiation. Furthermore, the other six *Melicope* species endemic to the Marquesas Islands would need to be included in a novel taxonomic recognition of Stone’s former sections *Megacarpa* and *Cubicarpa*.

## Data Availability

All demultiplexed raw read data were submitted to the NCBI Sequence Read Archive; BioProject number PRJNA559258.

## Author Contributions

MA, CP, and WW conceived and designed the study. MA, CP, and KW collected the samples. CP carried out the laboratory work and performed all analyses. DE provided valuable input for the analyses. CP drafted the manuscript, and all authors contributed to writing and editing. All authors have read and approved the final manuscript.

## Funding

This project was financially supported by the German Science Foundation (DFG; Grant AP 251/3-1 to MA). The funder had no role in study design, data collection and analysis, decision to publish, or preparation of the manuscript.

## Conflict of Interest Statement

The authors declare that the research was conducted in the absence of any commercial or financial relationships that could be construed as a potential conflict of interest.
