# Supplementary figures and images for: Phylogeny of Hawaiian Melicope (Rutaceae): RAD-seq Resolves Species Relationships and Reveals Ancient Introgression
Source: Front Plant Sci. 2019 Sep 17;10:1074. doi: 10.3389/fpls.2019.01074 (PMC6758601; doi:10.3389/fpls.2019.01074)

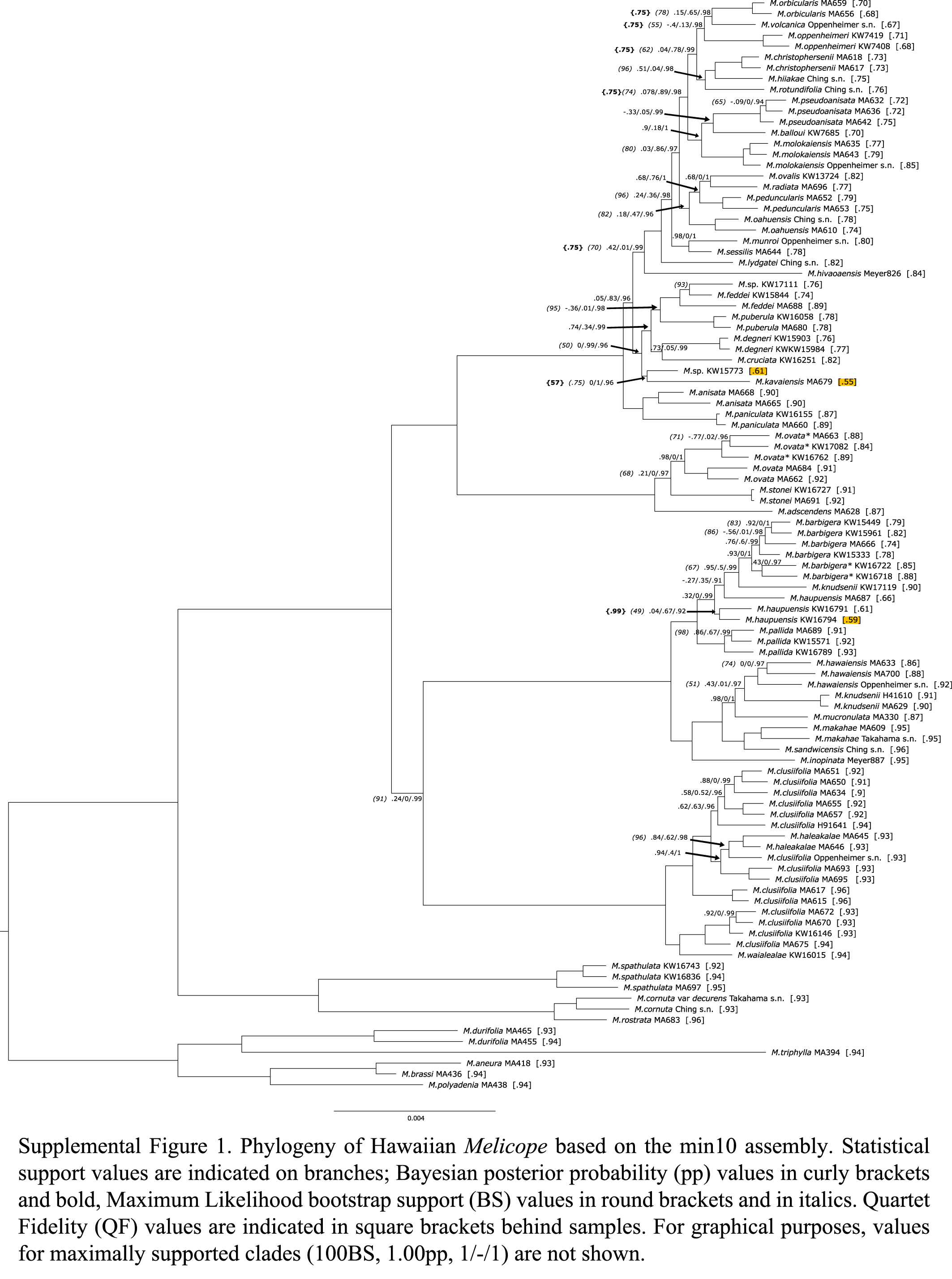

Supplement: Supplementary file 1 [file Image_1.tif]

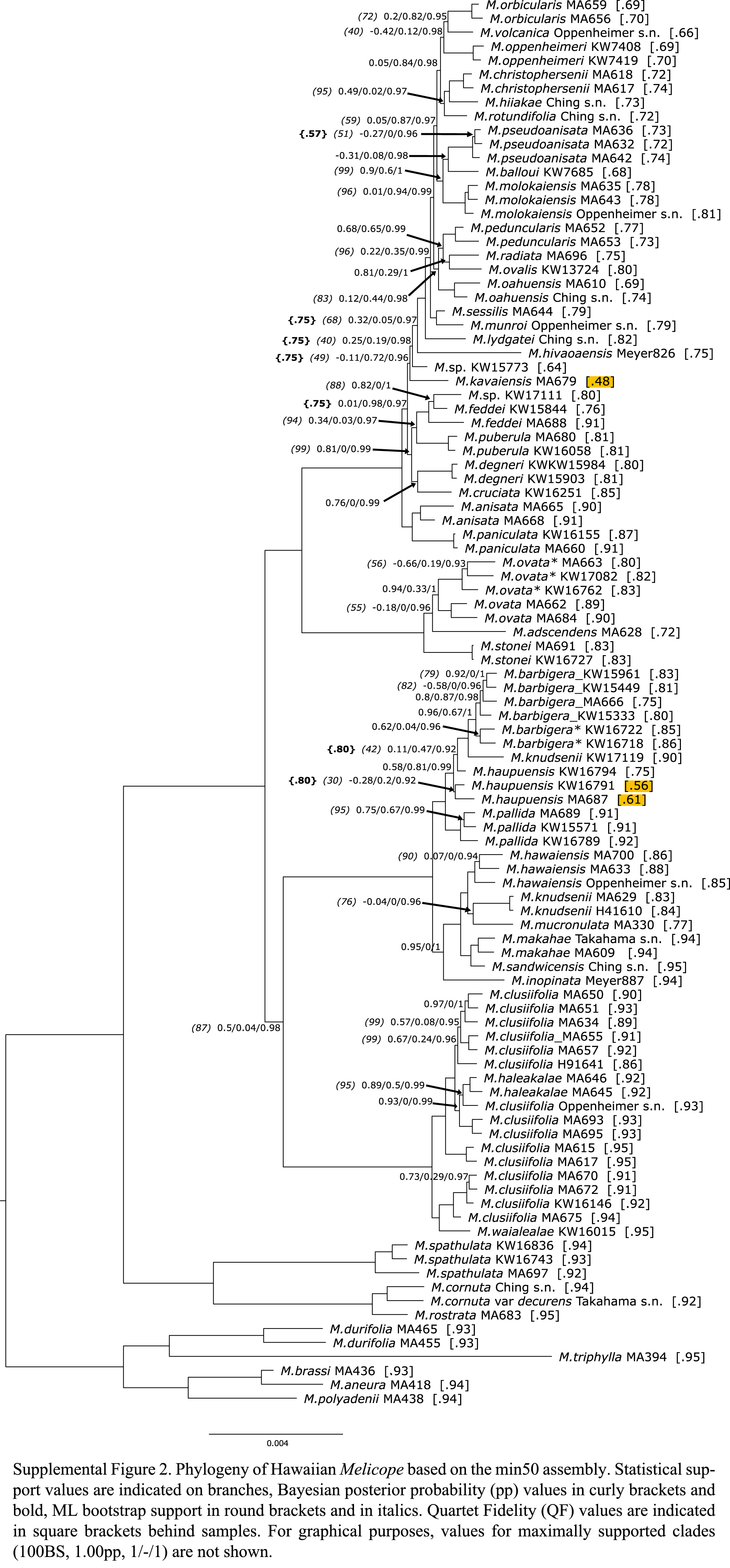

Supplement: Supplementary file 2 [file Image_2.tif]

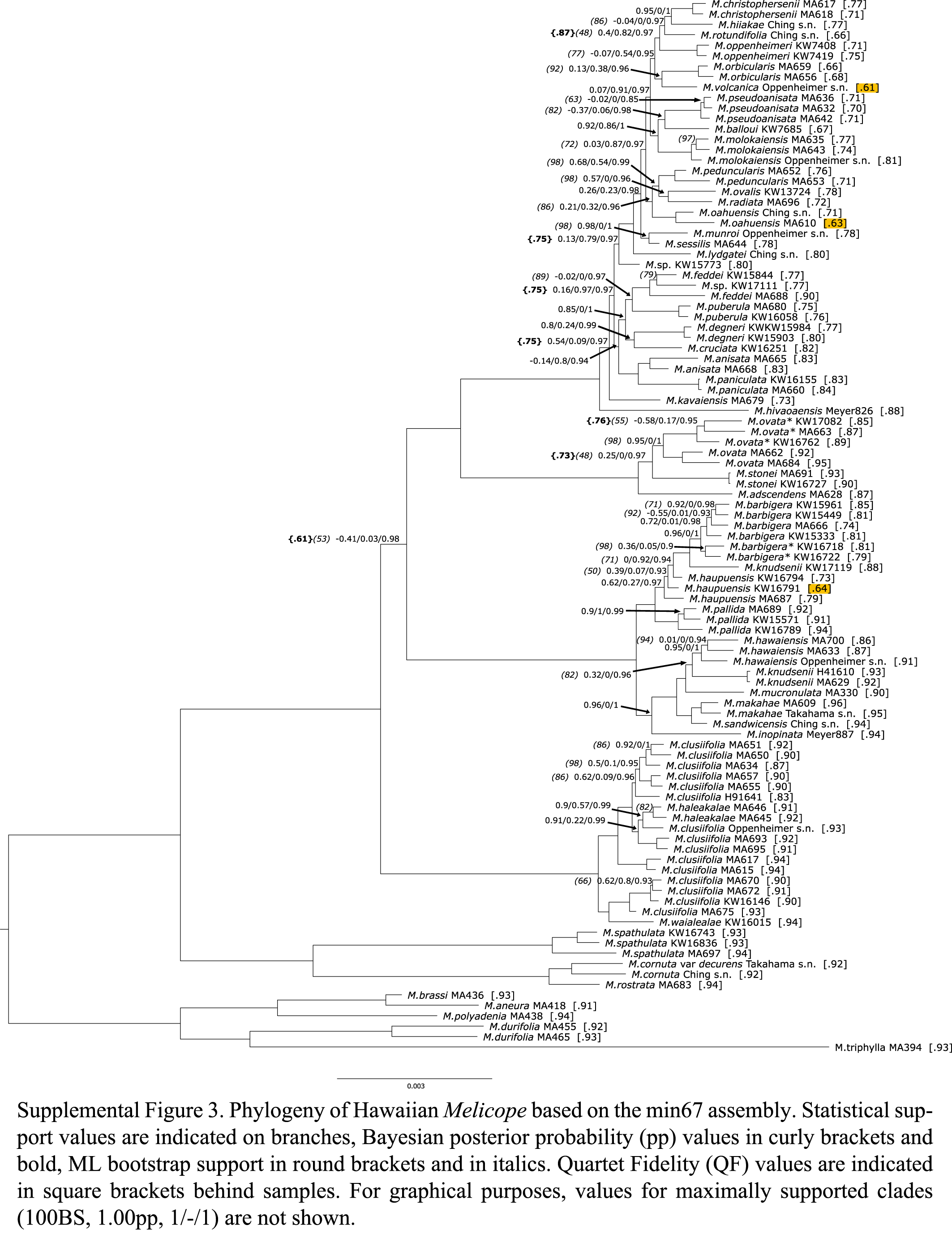

Supplement: Supplementary file 3 [file Image_3.tif]

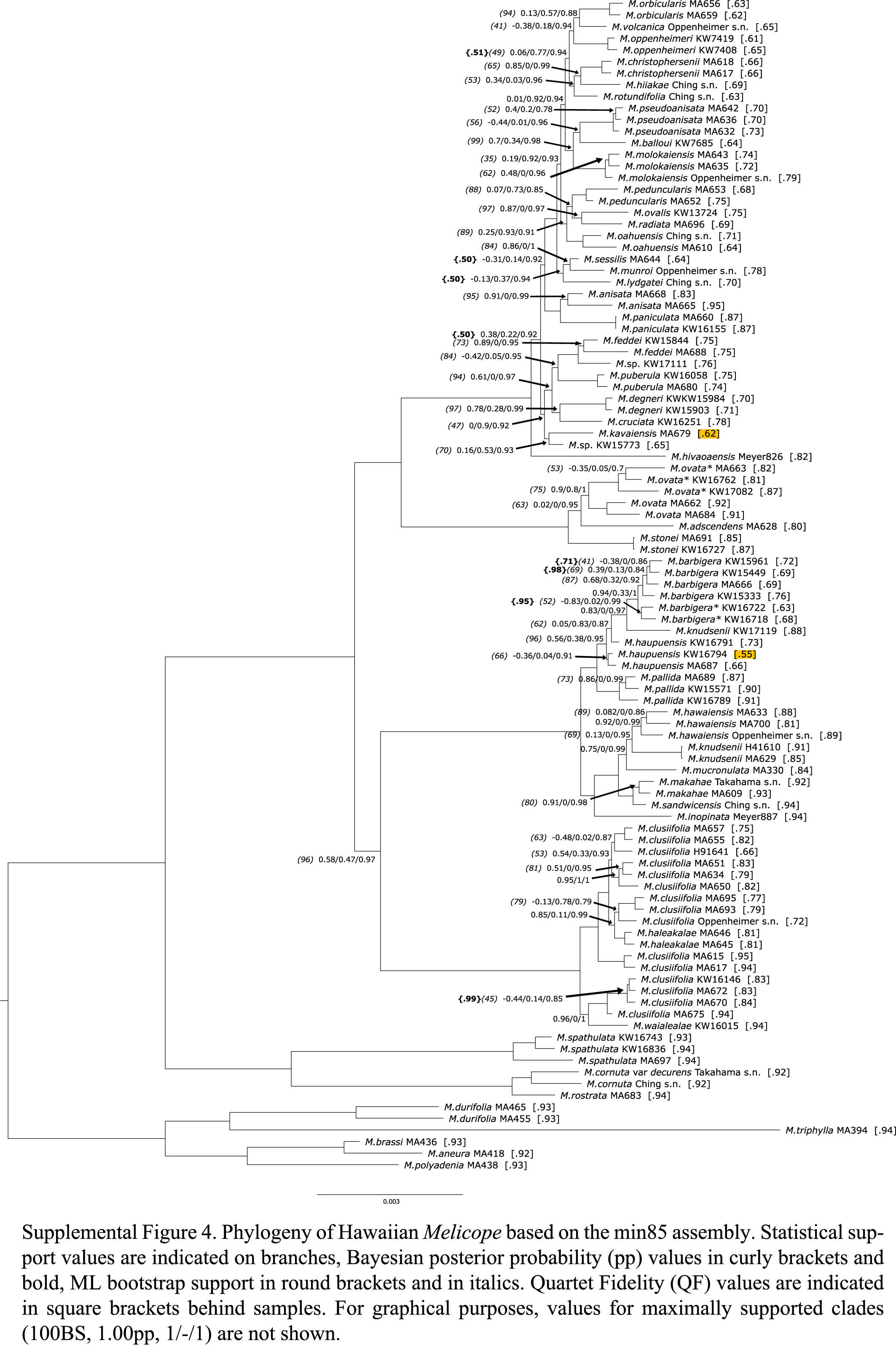

Supplement: Supplementary file 4 [file Image_4.tif]

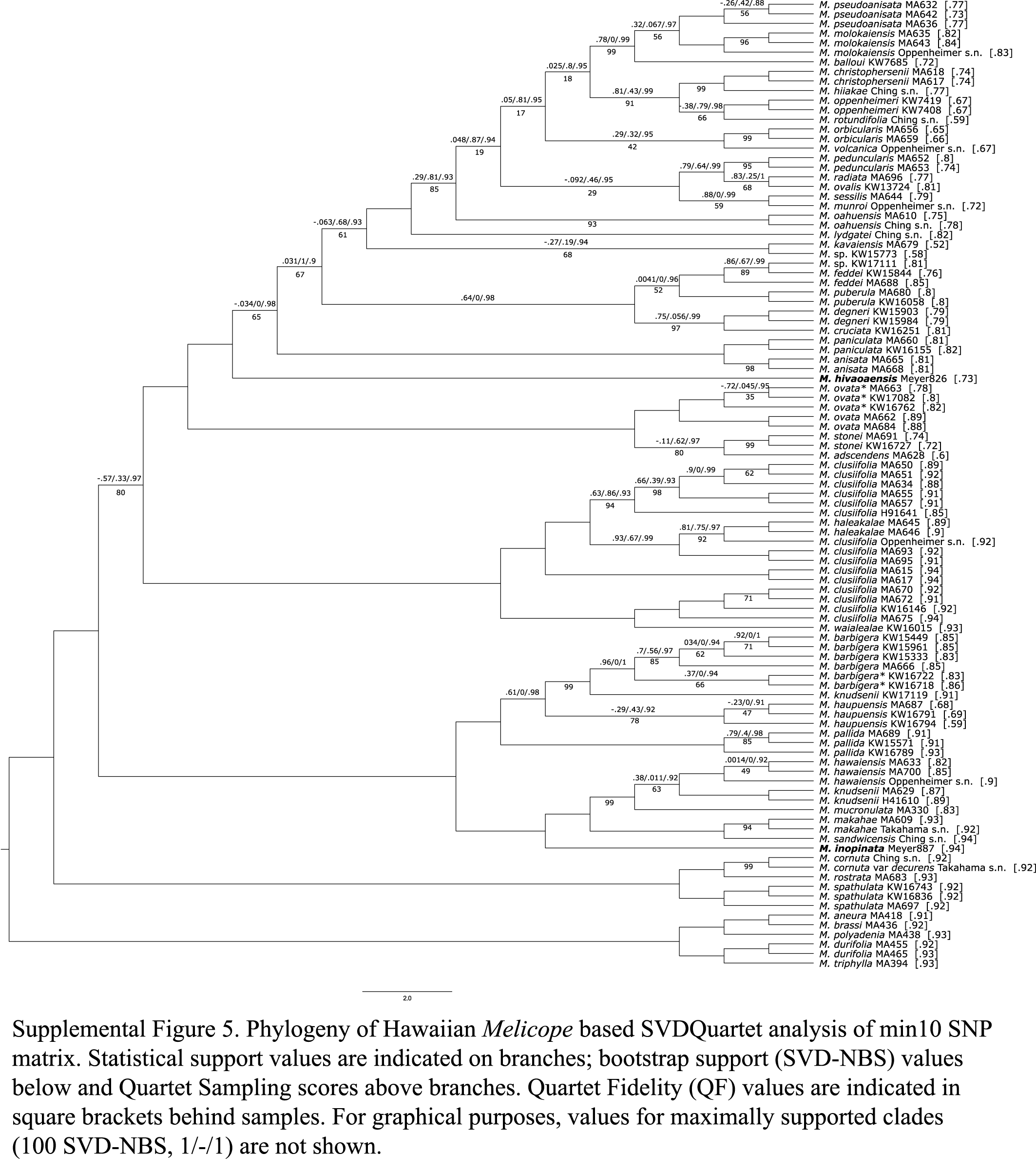

Supplement: Supplementary file 5 [file Image_5.tif]

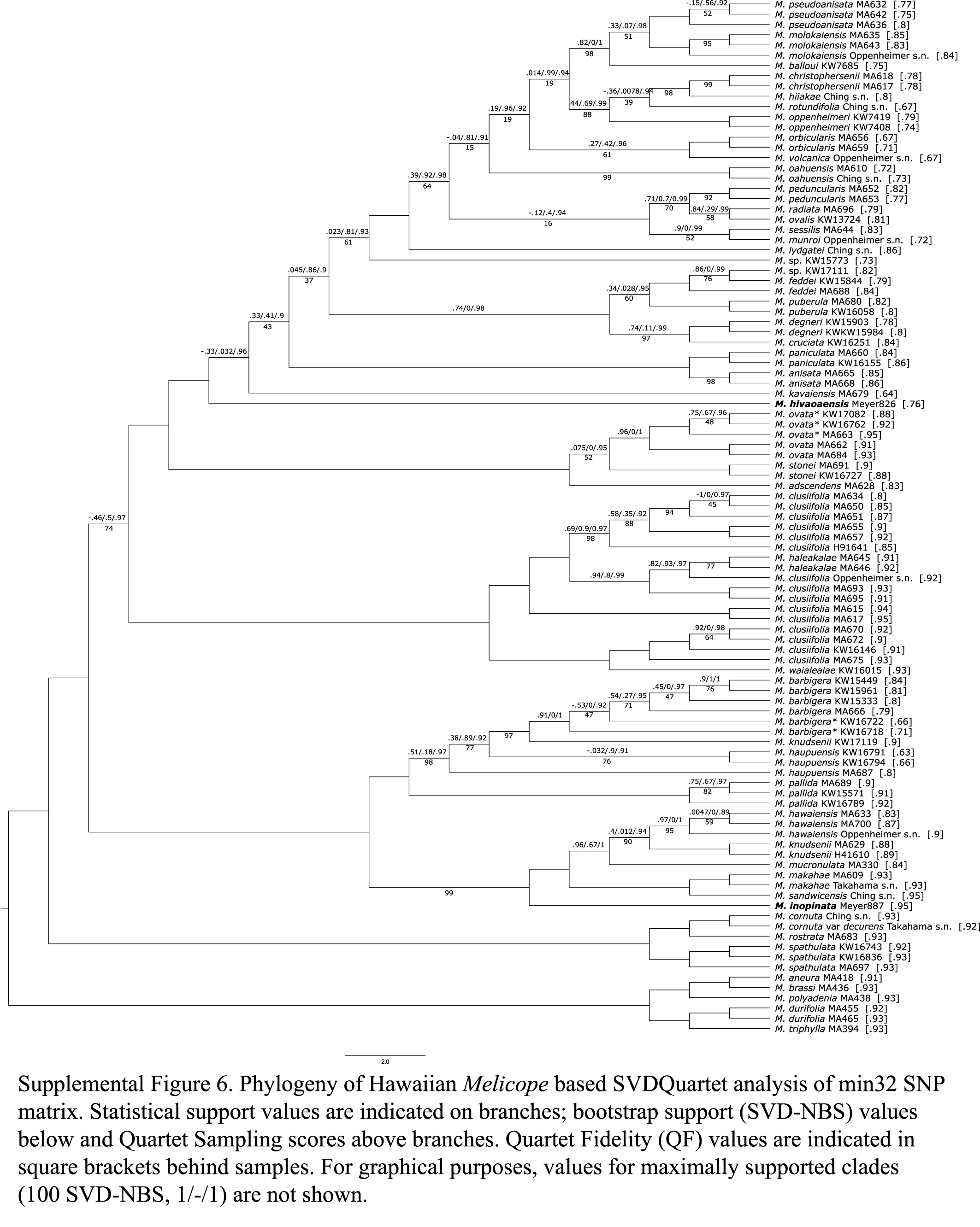

Supplement: Supplementary file 6 [file Image_6.tif]

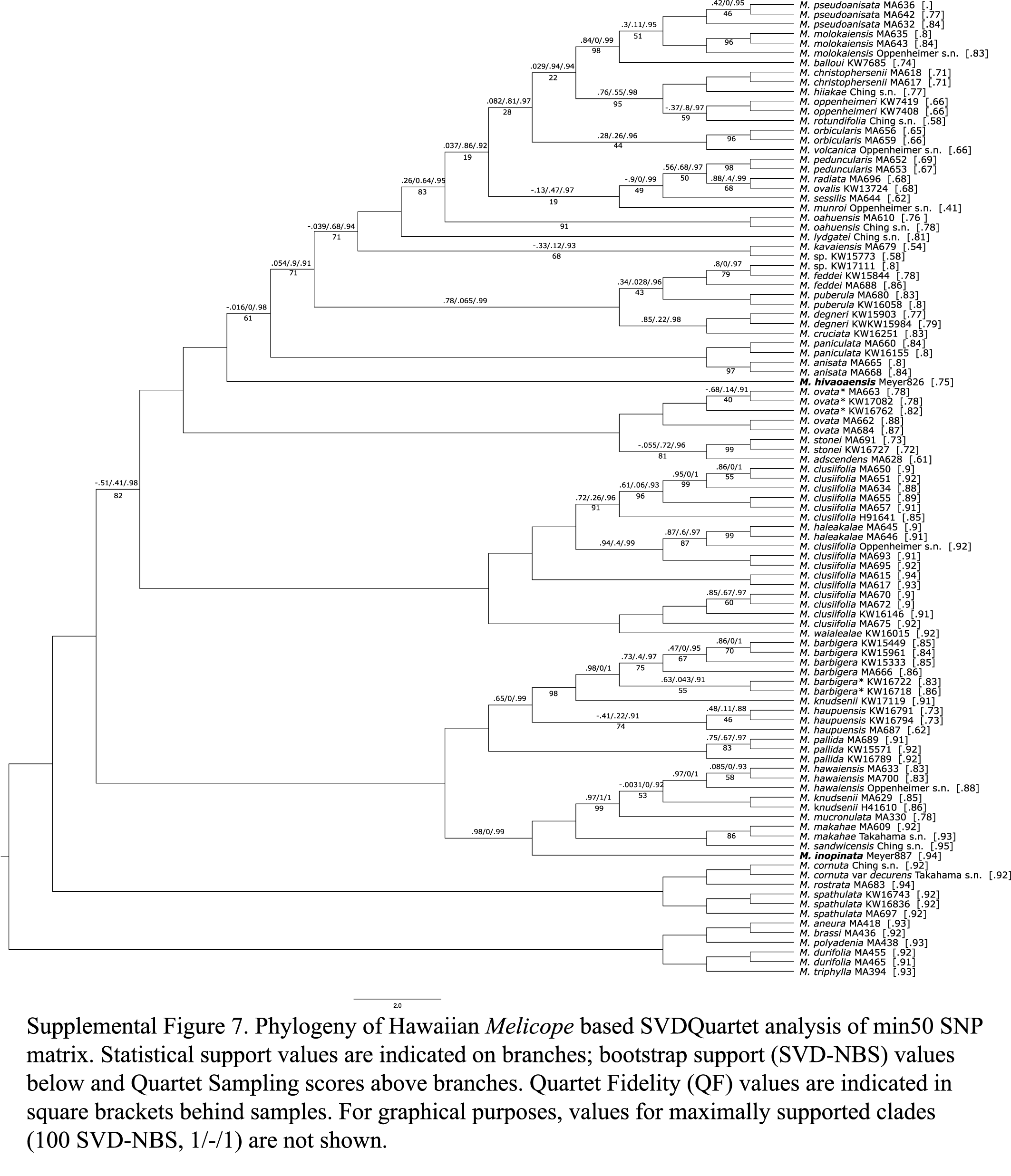

Supplement: Supplementary file 7 [file Image_7.tif]

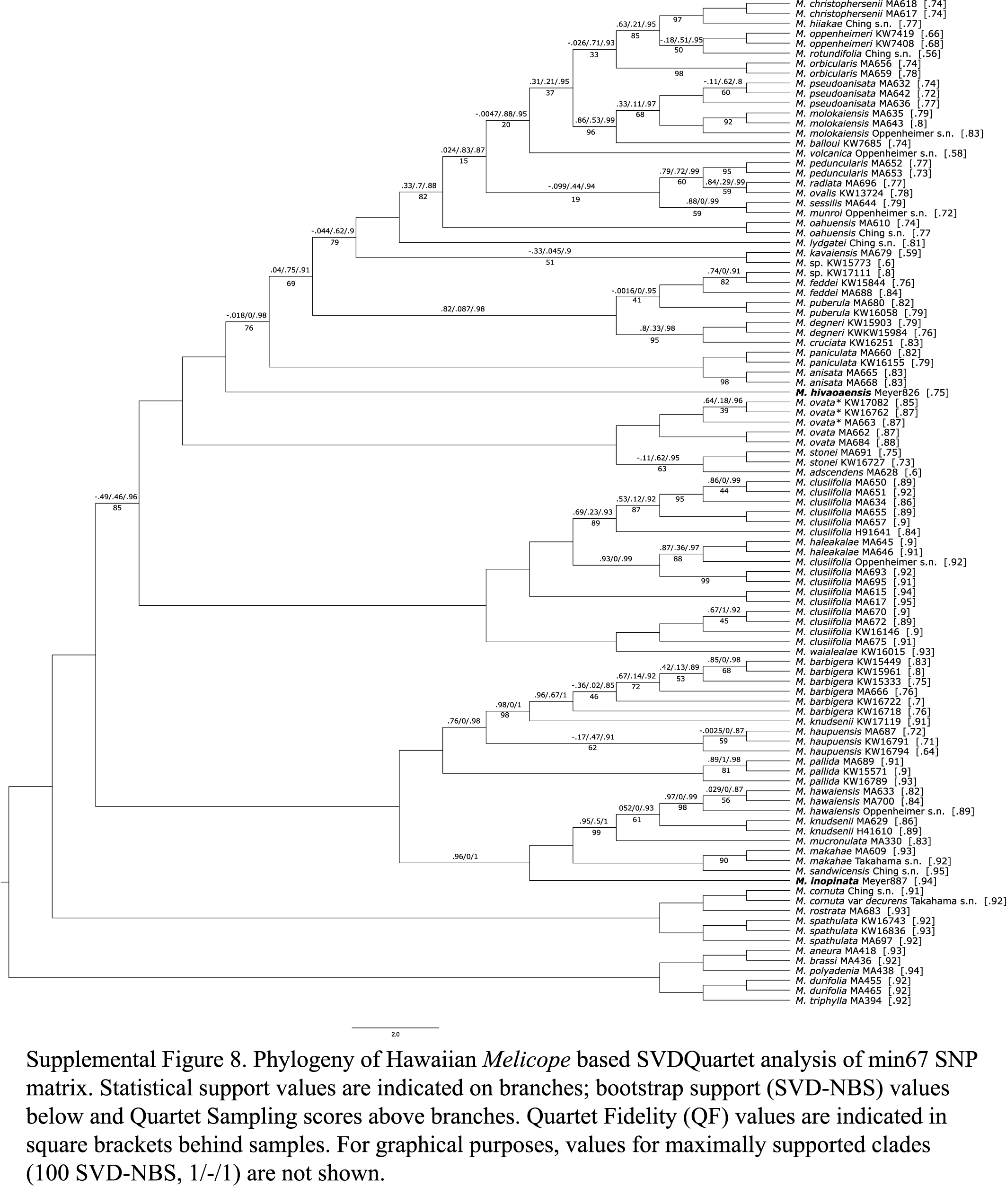

Supplement: Supplementary file 8 [file Image_8.tif]

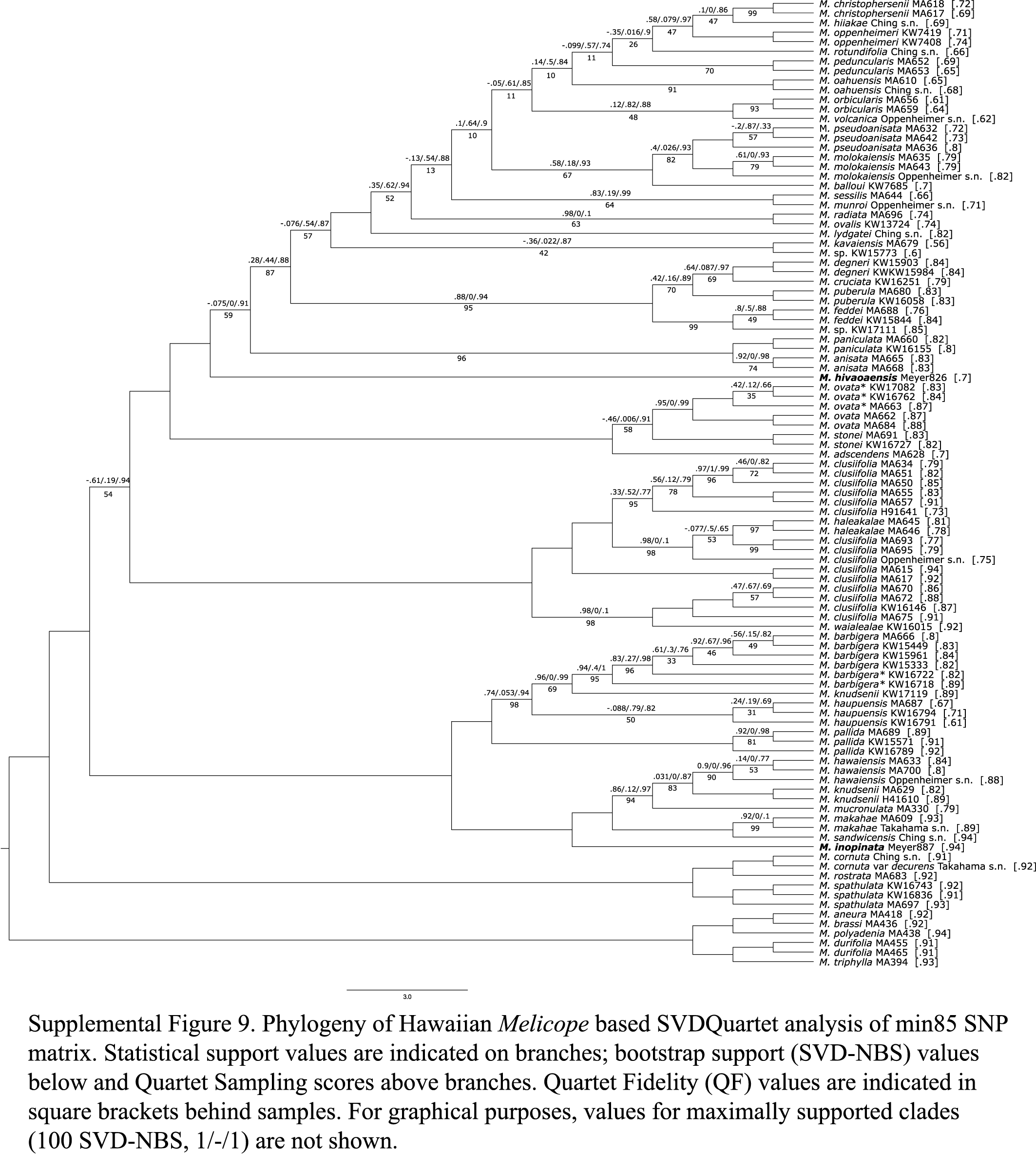

Supplement: Supplementary file 9 [file Image_9.tif]
